# Recent Update on siRNA Therapeutics

**DOI:** 10.3390/ijms26083456

**Published:** 2025-04-08

**Authors:** Oluwakemi Ebenezer, Abel Kolawole Oyebamiji, John Oludele Olanlokun, Jack A. Tuszynski, Gane Ka-Shu Wong

**Affiliations:** 1 Department of Physics, University of Alberta, Edmonton, AB T6G 2E1, Canada; re.korede@gmail.com; 2 Department of Industrial Chemistry, University of Ilesa, Ilesa PMB 5089, Nigeria; abeloyebamiji@gmail.com; 3 Laboratories for Biomembrane Research and Biotechnology, Department of Biochemistry, College of Medicine, University of Ibadan, Ibadan 200005, Nigeria; jodel72000@yahoo.com; 4 Department of Oncology, Cross Cancer Institute, University of Alberta, Edmonton, AB T6G 1Z2, Canada; 5 Department of Mechanical and Aerospace Engineering (DIMEAS), Politecnico di Torino, 10129 Turin, Italy; 6 Department of Data Science and Engineering, The Silesian University of Technology, 44-100 Gliwice, Poland; 7 Department of Biological Sciences, University of Alberta, Edmonton, AB T6G 2E9, Canada; gane@ualberta.ca

**Keywords:** siRNA therapeutics, clinical trial, diseases, tri-GalNAc conjugates, siRNA, gene therapy

## Abstract

Small interfering RNA (siRNA) has been deemed a promising therapeutic method for treating diverse diseases. siRNA-based therapeutics provide a distinct mechanism of action by selectively targeting and silencing disease-causing genes at the post-transcriptional level. This paper provides an overview of the present state of siRNA-based therapeutics, highlighting their potential in different therapeutic areas. The first section of this review introduces the basic principles of siRNA technology, including its mechanism of action and delivery methods. Subsequently, we discuss the impediments associated with siRNA delivery and manufacturing development and the strategies for overcoming these obstacles. The clinical advancement of siRNA therapeutics in various disease areas, including cancer, genetic disorders, viral infections, and inflammatory diseases, is summarized. Lastly, we summarize the successes, failures, and lessons learned from the development of siRNAs. With advancements in delivery systems and improvements in target selection, the field of medicine can be revolutionized, and siRNA therapeutics can offer new treatment options for patients.

## 1. Introduction

There is a longstanding interest in siRNA as a therapeutic modality due to its potential to selectively silence disease-causing genes with high specificity and potency. This contrasts with small molecules, where only about 15% of the genes can be targeted [1,2,3]. RNA interference is a naturally occurring biological process in cells that involves inhibiting gene expression at the post-transcriptional level. The discovery of RNA interference (RNAi) in the late 1990s by Andrew Fire and Craig Mello led to a new understanding of cellular mechanisms for gene regulation [4]. It opened up opportunities for developing RNA-based therapeutics. Its application was initially impeded because double-stranded RNAs (dsRNAs) of varying lengths activate the interferon response, resulting in a non-specific reduction in messenger RNA (mRNA) levels. To resolve this problem, researchers found that dsRNA fragments with 3′ overhangs of two nucleotides can efficiently degrade sequence-specific mRNA [5,6]. These small interfering RNAs are usually double-stranded RNA molecules, typically 21–23 nucleotides long, and are designed to complement the target mRNA sequence [7,8,9,10]. As a result, these siRNAs can impede the expression of targeted genes in mammalian cells without triggering interferon production. siRNA drugs have emerged as a promising class of therapeutics in the field of molecular biology and gene regulation. These small RNA molecules can bind to messenger RNAs (mRNAs) and interfere with their translation into proteins or induce their degradation, thereby controlling gene expression [11,12,13,14]. Inspired by the therapeutic potential of RNA interference, researchers began developing methods to exploit this mechanism for targeted gene silencing. siRNA drugs are designed to specifically silence disease-causing genes by introducing synthetic siRNA molecules into cells. The introduction of siRNAs triggers a series of enzymatic reactions in the cell to form RNA-induced silencing complexes (RISCs). The RISCs bind to the target mRNA and guide its degradation, preventing the production of the corresponding protein [15,16]. Moreover, siRNA therapeutics can potentially address diseases previously considered challenging to treat [17,18]. This opens up new possibilities for tackling genetic disorders as well as various types of cancers, viral infections, inflammatory diseases, and neurodegenerative diseases. They can also be used to study gene function in research settings [19,20,21,22].

In practice, drug delivery and manufacturing considerations are involved. It is never an easy task to get a drug to where it is needed and have it get there at the required concentration. The challenges are even more significant because of the peculiarities of siRNA. The development of siRNA drugs has faced challenges, particularly in terms of delivery methods. Efficient delivery and cellular uptake of siRNAs remain critical for successful therapeutic outcomes. Many delivery cargos, such as lipid nanoparticles, viral vectors, and conjugated molecules, have been explored to enhance siRNA stability, cellular uptake, and target specificity.

Despite these challenges, some siRNA drugs have now been approved, and we are beginning to get a sense of what this therapeutic modality is promising for and what it is not suitable for. Ongoing research continues to explore the therapeutic potential of siRNA drugs, aiming to improve their specificity, stability, delivery, and safety profiles. Additionally, many delivery systems with promising outcomes have been investigated, namely Local Drug EluteR (LODER™) polymers, Targeted RNAi Molecule (TRiM™), Lipid nanoparticles (LNPs), Dynamic polyconjugates (DPC™), *N*-acetylgalactosamine (GalNAc)–siRNA conjugates, exosomes, and polypeptide nanoparticles (PNPs). In 2018, the first RNAi-based therapeutic, Onpattro (patisiran), was approved by the U.S. Food and Drug Administration (FDA) to treat hereditary transthyretin amyloidosis, a rare genetic disease. This approval marked a significant milestone in the field of drugs, highlighting their potential for treating genetic disorders. Thus, in this review, we discuss (i) The mechanism of action of siRNA, (ii) The challenges in siRNA and the way out, (iii) Manufacturing challenges, (iv) Naked, LNPs, and GalNAc–siRNA conjugate therapeutic agents, and (v) Successes, failures, and lessons learned so far from the development of siRNAs. It can be concluded that the development of siRNA drugs holds promise for personalized medicine and targeted therapies, and it will offer new treatment options for various diseases.

## 2. Mechanism of Action of RNA Interference

**Figure 1:**
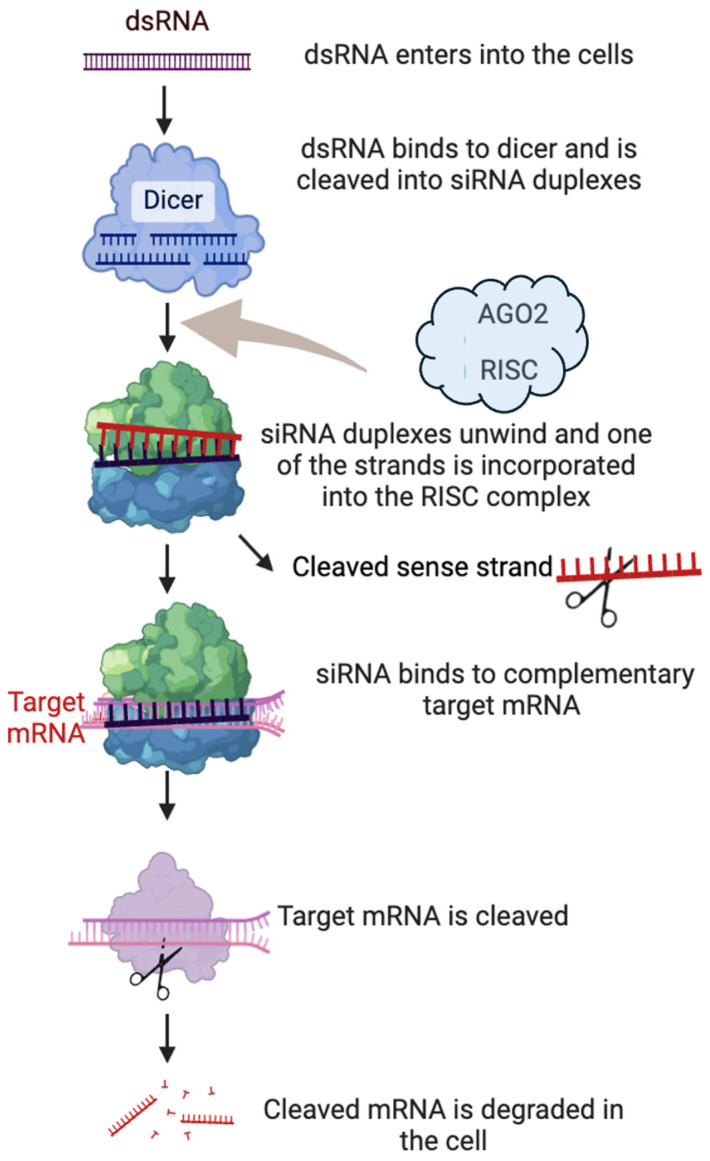
Gene silencing mechanisms of RNAi. The RISC loads siRNA generated by Dicer from dsRNA. An enzyme called AGO2 separates the passenger strand of siRNA from the guide strand. RISC then binds to target mRNA through the guide strand, which leads to mRNA cleavage through complementary binding between siRNA and target mRNA. The icons were created using Biorender.com.

RNA interference is a natural cellular process that targets mRNA for degradation, and this would result in silencing of gene expression. It plays a vital role in gene regulation and innate defense against invading viruses. As previously discussed, siRNA exerts its effects at the post-transcriptional level as a 21–23 nucleotide-long double-stranded RNA molecule. The first step of RNAi involves the recognition and cleavage of long double-stranded RNA. Dicer recognizes and binds to long double-stranded RNA molecules, such as synthetic siRNA or precursor miRNA. The siRNA duplex comprises a passenger (sense) and a guide (antisense) strand. In the subsequent step, referred to as loading into the RISC, the dicer-processed siRNA fragments are loaded into the RISC complex (Figure 1). Within the RISC complex, siRNA strands are separated, and the strand with the more stable 5′-end is typically integrated into the active RISC complex. The endonuclease argonaute 2 (AGO2) component of the RISC cleaves the siRNA’s passenger strand (sense strand), resulting in duplex unwinding and degradation of the passenger strand. In contrast, the guide (antisense strand) remains associated with the RISC.

Lastly is the mRNA target recognition; the siRNA-loaded RISC complex scans cellular mRNA molecules to find a target mRNA complementary to the siRNA guide strand. Once a complementary target mRNA is found, the RISC complex induces cleavage of the mRNA at a specific site, leading to its degradation and inhibition of gene expression [9]. Indeed, it has been reported that long double-stranded RNA molecules, including siRNAs longer than 30 nucleotides, can trigger an innate immune response in mammalian cells, leading to the induction of interferons. This response is part of the cellular defense mechanism against viral infections, as viruses often produce long double-stranded RNA molecules during their replication process. Additionally, when designing siRNAs for experimental or therapeutic applications, it is crucial to identify the lowest concentration at which a particular siRNA can induce effective gene silencing [23] and thus keep the length within the optimal range to avoid triggering an unwanted immune response.

## 3. Challenges and the Way out

**Figure 2:**
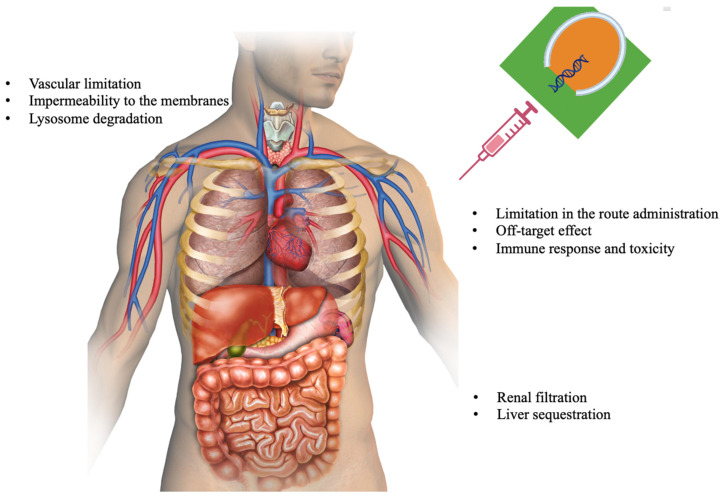
Challenges to be overcome in the siRNA therapeutics.

SiRNA has emerged as a promising tool in the field of therapeutic applications. This small, double-stranded RNA molecule could silence specific genes, offering potential treatments for various diseases. While siRNA technology provides many advantages over traditional small molecule therapeutics, some challenges need to be addressed, such as delivery efficiency, stability, immune stimulation, and potential off-target effects (Figure 2). Ongoing research and development efforts are focused on overcoming these challenges to harness the full potential of siRNA technology for treating a wide range of diseases. The successful application of siRNA therapeutics in diverse disease conditions can be achieved by addressing these challenges through ongoing research and technological advancements.

### 3.1. siRNA Delivery

The main challenge with RNAi technology’s therapeutic application is how it is delivered. siRNA must be transported through the body without clearance or degradation to take effect in the cytoplasm of a target cell. The safety and efficient delivery of siRNA, to a certain extent, is influenced by the capability of the siRNA to dive into the target organ or tissue. The bioavailability of the drug will be facilitated by siRNA therapy’s ability to successfully reach the specified target (local delivery). In the last few decades, there have been reports of various non-viral carriers that are gaining more and more attention as reliable and effective ways to deliver siRNAs to targeted organs. Such as physical methods (hydrodynamic injection particles, electroporation, and bombardment) and biochemical processes [24,25,26,27,28,29,30]. Additionally, many researchers have explored polymers, including proteins and peptides, as delivery vehicles for siRNA [30]. The challenges with siRNA systemic administration include poor bioavailability, rapid excretion, systemic toxicity, and ineffective targeting of the affected organ or cell type [31,32]. Numerous studies have examined various approaches to local delivery of therapeutic siRNA. This route has become desirable and practical due to lower doses and the minimal side effects. Strategies to overcome this challenge include using viral vectors, lipid nanoparticles, chemical modifications, or the utilization of tri-GalNAc conjugates for precise oligonucleotide delivery to hepatocytes, which has emerged as a significant advancement in the field of therapeutic oligonucleotides [33,34,35,36].

### 3.2. Off-Target Effects

Despite the effort to deliver siRNAs to specific targets, studies have shown the possibility of the siRNAs going off-target by silencing untargeted genes. The innate immune response can produce off-target effects through post-transcriptional gene inhibition, but siRNA-induced miRNA-like impacts are considered the potential contributor [37]. The miRNA-like impact might, therefore, be mediated by one of two alternative mechanisms: either using siRNAs’ promiscuous access to the endogenous miRNA machinery [38] or through the sequence-dependent regulation of undesirable transcripts, i.e., through their 3′UTRs’ partial sequence complementarity [39]. The off-target effect is associated with in vivo RNAi, resulting in unintended biological consequences, making it challenging to interpret siRNA’s therapeutic effectiveness and jeopardizing RNAi treatment’s safety. Efforts are being made to design and select siRNA molecules that are extremely specific to the target gene to minimize off-target effects. It is crucial to overcome the off-target effects, which are possible by chemical alteration of nucleotides surrounded by the seed area. For instance, replacing a 2′-O-Me at a specific nucleotide spot in the seed region is sufficient to suppress and inactivate the off-target movement of the siRNA without significantly harming the silencing of the proposed targets [40,41]. The siRNA-induced resistant initiation can be reduced by substituting uridines with modified 2′-Ome, 2′-F, and 2′-deoxy homologs. It has been reported that these changes repeal the immune identification of siRNAs by TLRs [42].

### 3.3. Bio-Stability

The chemical alteration that lengthens siRNA half-life without endangering biological activity has been examined as a way to turn siRNAs into enhanced medicines. Better nuclease stability is critical to expose siRNA duplexes to nuclease-rich surroundings (like blood) [43,44]. Also, when administering siRNA directly to sites less enriched in nucleases, the level of nucleases’ stabilization can be considerably diminished when siRNA is distributed in conjunction with transport vehicles, like liposomes, or organs, such as the lung [45]. Introducing different chemical changes can potentially enhance the stability of siRNA nucleases. To retain the siRNA cleavage activity during the modifications, phosphate or a free hydroxyl group must be preserved at the 5′ end of the antisense strand [42]. The change of internucleotide phosphate by substitution of the phosphodiester functional group with phosphorothioate (PS) at the 3′ end linkage of the RNA strands can impede enzymatic degradation by lessening the vulnerability of siRNAs to exonucleases, resulting in nuclease resistance [46].

Further, the stability of siRNA can be significantly improved by altering the 2′-position of ribose to reduce the vulnerability of internucleotide phosphate linkage to nuclease cleavage and lengthen their half-life. The introduction of locked nucleic acid (LNA) can substantially impact the half-life of siRNAs by enhancing nuclease stability and stabilizing the duplex structure [47]. Ensuring stability and protection of siRNAs during delivery and within cells is crucial for their therapeutic efficacy. Various chemical modifications or encapsulation strategies are being explored to enhance stability. In addition, numerous studies on the chemical modifications of siRNA molecules have been published [48,49,50,51], and these have been summarized in Table 1.

### 3.4. Immunogenicity

siRNA molecules can trigger an immune response in the body. This occurs because the immune system recognizes double-stranded RNA (dsRNA) molecules, such as siRNA, as a sign of viral disease. The immune response can activate various immune cells, release inflammatory mediators, and produce cytokines. The immunogenicity of siRNA can have several implications. Firstly, it could lead to unwanted results and adverse patient reactions, such as flu-like symptoms, fever, or infusion-related reactions [52]. These immune reactions can limit the tolerability and safety of siRNA therapeutics. Secondly, the immune response can reduce the therapeutic efficacy of siRNA molecules. Activation of the immune system can lead to the rapid degradation or clearance of siRNA molecules, thereby reducing their concentration and duration of action in the target cells or tissues [53]. Additionally, the immune response can trigger the production of interferon, which can interfere with the RNAi pathway and disrupt the gene-silencing effect. Several strategies are being employed to address the challenge of immunogenicity. One approach is to design siRNA molecules with specific structural modifications that can reduce immune activation. For example, specific chemical alterations to the sugar hybrid or nucleotide bases of siRNA can enhance stability and minimize immune recognition [48,54,55]. Another approach is to develop appropriate delivery systems that can shield siRNA molecules from the immune system. Various nanoparticle-based delivery systems, namely lipid nanoparticles or polymer-based (synthetic or natural) carriers, can protect siRNA from degradation and minimize immune recognition [56,57,58,59,60]. Additionally, meticulous selection and design of siRNA sequences can minimize their immunogenic potential. Extensive screening and optimization processes are employed to identify siRNA molecules that are less likely to trigger an immune system. Tackling the immunogenicity of siRNA molecules is crucial because this will enhance their clinical success. Therefore, researchers aim to improve siRNA’s safety, efficacy, and therapeutic potential to minimize immune activation and enhance stability.

### 3.5. Limited Tissue Penetration

siRNAs often face challenges penetrating specific tissues or barriers, such as the blood-brain barrier. It is well evident that siRNAs are large and negatively charged molecules. These properties make it difficult for them to efficiently cross biological barriers and reach target cells in specific tissues. We should all understand that the extent of tissue penetration depends on various factors, such as the route of administration, the delivery system used, and the specific tissue or organ being targeted. For example, the liver and kidneys often accumulate siRNAs from systemic administration, as these organs have a high uptake capacity for nucleic acids [61,62]. However, other tissues like the brain, heart, or lungs may have limited siRNA uptake [63]. To overcome this challenge, researchers have been developing various strategies to enhance tissue penetration of siRNAs. One approach is the use of targeted delivery systems. These include nanoparticles with surface modifications that can specifically identify and bind to receptors or fragments of molecules on the target cells, enabling efficient uptake [64,65]. This can improve siRNA transport to the exact tissues or cell types. Another strategy involves the use of physical methods to enhance tissue penetration. For example, electroporation consists of applying electric pulses to enhance the permeability of cell membranes, permitting siRNA to enter target cells more efficiently [66,67,68]. Similarly, ultrasound-mediated delivery techniques use sound waves to temporarily disrupt cell membranes and promote siRNA uptake [69,70]. Engineering siRNAs with chemical modifications can also help enhance tissue penetration. Specific alterations to the siRNA structure can increase stability, reduce degradation, and improve cellular uptake. For example, lipid conjugation or lipid-based formulations can improve siRNA delivery and facilitate tissue penetration [71,72,73,74]. Furthermore, advances in carrier systems, such as exosomes or cell-penetrating peptides, are being explored to enhance siRNA delivery and improve tissue penetration [75,76,77,78]. These carriers can efficiently transport siRNAs across biological barriers and facilitate their uptake by target cells. Although limited tissue penetration is a crucial challenge for siRNA therapeutics, ongoing research and advancements in targeted delivery systems, physical methods, chemical modifications, and carrier systems offer promising solutions to enhance tissue penetration and improve the effectiveness of siRNA-based treatments.

### 3.6. DNA and RNA Modification

RNA modifications are crucial in regulating gene expression and can significantly impact siRNA stability, specificity, and therapeutic efficacy. N^6^-methyladenosine (m^6^A), the most prevalent internal mRNA modification, exerts regulatory control over RNA metabolism, specifically affecting degradation, translation, stability, and export, and thus mediates diverse physiological and pathological outcomes, including stress adaptation, immune modulation, developmental programming, and oncogenesis [79,80,81]. This modification could affect siRNA-mediated gene silencing by altering target mRNA accessibility or stability. Similarly, 5-methylcytosine (m^5^C) modifications in RNA can affect its structure and function, potentially impacting siRNA binding and efficacy [82,83,84]. Research has shown that there is crosstalk between m6A and 5mC regulation, which means that changes in one can influence the other. This, in turn, could cause a compounded indirect effect on siRNA activity [85,86]. Optimizing siRNA therapeutics demands a consideration of the interplay between DNA and RNA modifications. Breakthroughs in sequencing and computational analysis of RNA modifications provide a pathway to enhanced siRNA efficacy [79,81,83,84,87,88]. These methods enable the prediction of modification sites on target mRNAs, facilitating precise siRNA targeting and minimizing off-target effects. Moreover, elucidating the dynamic distribution of RNA modifications across cellular contexts is vital for developing strategies to improve siRNA stability and delivery. As our understanding of RNA modifications deepens, the evolution of siRNA design principles will enable the creation of more sophisticated and disease-specific RNA interference therapies, offering new avenues for treating cancer and cardiovascular disorders.

## 4. Manufacturing Challenges of siRNA

**Figure 3:**
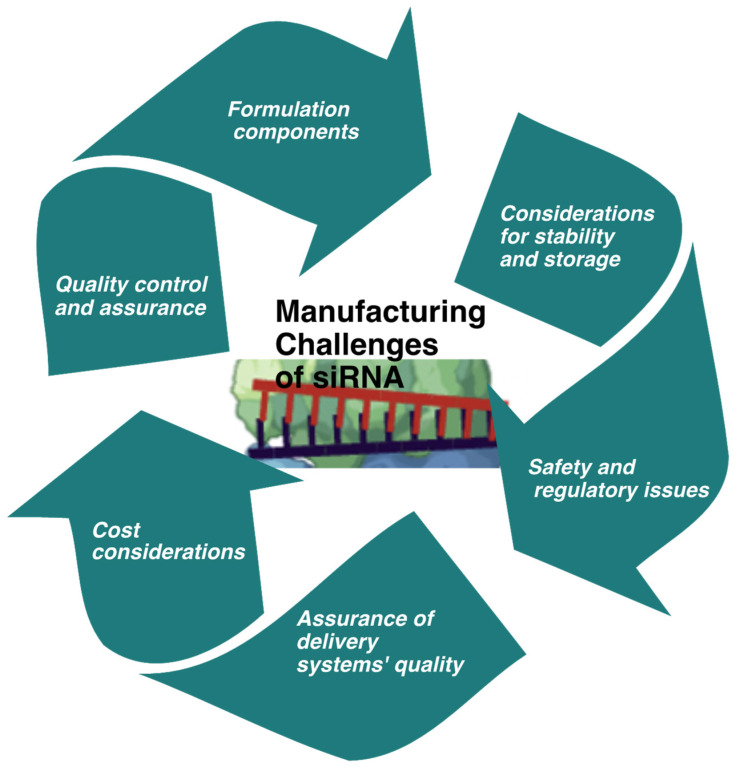
Manufacturing challenges of siRNA therapeutic agents.

The manufacturing process for siRNAs typically involves chemical synthesis, including solid-phase or enzymatic methods. The large-scale production and manufacturing of siRNA therapeutics can be complex and expensive. Developing efficient and cost-effective manufacturing processes will make siRNA therapeutics more accessible to patients. Solid-phase synthesis is the most common method and involves sequentially adding nucleotides to a growing chain anchored on solid support [89,90,91]. This process can be automated and allows for high-throughput synthesis of siRNAs. Enzymatic methods, such as in vitro transcription, can produce siRNAs, although they are less commonly used [92,93]. Notably, the cost of siRNA manufacturing can be influenced by factors such as the production scale, the purity and quality requirements, and any additional modifications or conjugations required. Large-scale synthesis can help reduce costs due to economies of scale. Additionally, advancements in synthesis technologies and processes have improved the efficiency and reduced the cost of siRNA production over the years (Figure 3). However, despite these advancements, the cost of manufacturing siRNAs can still be relatively high compared to other alternative therapies. This is primarily due to the complex and sophisticated synthesis procedures involved, the need for high purity and quality control, and the cost of raw materials. For example, drugs like patisiran, givosiran, lumasiran, vutrisiran, and nedosiran, used for rare genetic conditions, range from roughly USD 400,000 to USD 1,640,000 annually [94,95,96,97,98]. While their alternative therapies may have lower direct drug costs, they often involve complex and expensive procedures (like organ transplants) or substantial ongoing costs for specialized care and management. Inclisiran, for high cholesterol, is a notable exception with a much lower annual cost of around USD 3250, competing with other lipid-lowering medications. Although the cost of siRNA manufacturing can be relatively high, technological advancements and optimization strategies offer opportunities to reduce expenses and make siRNA therapies more accessible and affordable.

Here are some key considerations:

***Quality control and assurance***: The safety and effectiveness of siRNA-based therapies depend on maintaining stringent quality control and assurance standards throughout the production process, primarily due to the complex nature of these molecules and their delivery systems [99]. This includes rigorous testing and characterization of siRNA products to ensure their purity, stability, and appropriate endotoxin levels. Implementing comprehensive quality management systems and adhering to Good Manufacturing Practices (GMP) are vital to ensure product quality, consistency, and reliability.

***Considerations for stability and storage:*** siRNA molecules may be susceptible to changes and degradation that could reduce their effectiveness [100]. The development of nanoparticle-based delivery systems for siRNAs presents its own set of manufacturing challenges. These nanocarriers must be produced with consistent size, shape, and surface area modifications to ensure reproducible pharmacokinetics and biodistribution [101]. Additionally, stability studies are necessary to establish ideal storage conditions and shelf life for siRNA-based therapies. Freeze-drying or encapsulating siRNA molecules in stabilizing chemicals may be required to increase their stability during storage and transit.

***Assurance of delivery systems’ quality:*** Delivery systems must adhere to specific quality criteria in addition to siRNA molecules to transfer siRNAs to target cells or tissues. To guarantee the delivery systems’ suitability for siRNA therapies, the manufacturers must prove the systems’ security, effectiveness, and stability and conduct rigorous risk analyses.

***Cost considerations:*** Scalability and cost-effectiveness are essential for widely adopting siRNA-based therapeutics. Manufacturers must develop efficient production processes and optimize costs without compromising quality and safety.

Collaboration between industry, academia, and regulatory authorities is essential to create a favorable regulatory environment and establish clear guidelines for manufacturing processes, quality control, and product evaluation to ensure the safe and effective use of siRNA therapeutics. The prospects of siRNA-based therapeutics are promising and hold great potential for advancing the field of medicine. One of the primary obstacles in siRNA therapeutics is efficient and targeted delivery to the desired cells or tissues. Ongoing research focuses on developing improved delivery vehicles, such as lipid nanoparticles, viral vectors, exosomes, and conjugate-based approaches. The enhancement of the delivery systems will increase the siRNA stability, improve cellular uptake, and enhance the targeting of specific tissues. It is worth mentioning that identifying the correct targets for siRNA therapy is crucial. This could involve using advanced genomics, proteomics, and computational approaches to identify disease-specific targets with high therapeutic potential. Further, the combinations of siRNA-based therapeutics with other treatment modalities, such as small molecule drugs, antibodies, or immune checkpoint inhibitors, hold promise for synergistic effects and could improve therapeutic outcomes. Combinatorial approaches may enhance the treatment efficacy, overcome drug resistance, and provide personalized therapeutic options. Additionally, siRNA-based therapeutics can also be combined with gene editing technologies like CRISPR-Cas9 to achieve precise modifications in the genome [102]. The prospects of siRNA-based therapeutics are exciting, with ongoing efforts to improve delivery systems, target selection, combination therapy approaches, and manufacturing processes. The advancements in these areas are expected to lead to the development of safe and effective siRNA-based treatments for various diseases, offering new options for patients and potentially transforming the landscape of modern medicine. Optimization and focus on the delivery of cargo are areas that need future attention, as well as the preferable administration route by the patients.

## 5. Naked, LNPs, and GalNAc–siRNA Conjugate Therapeutic Agents

**Figure 4:**
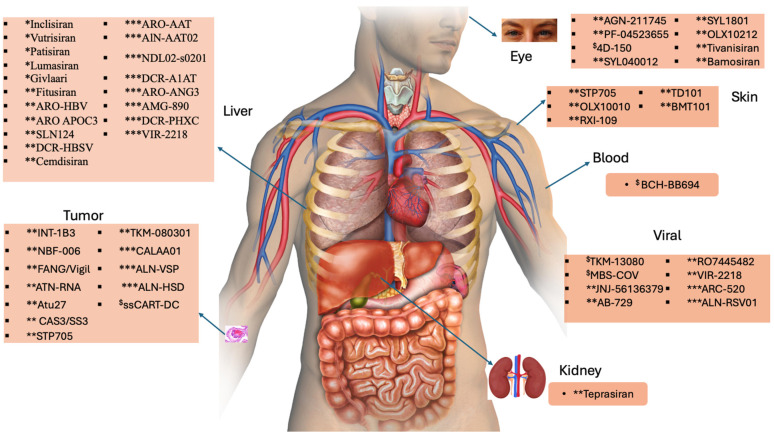
Presentation of siRNA therapeutics that are currently in the preclinical trial (^$^), approved (*), clinical trial (**), and discontinued (***) inspired by therapeutic siRNA: state of the art, Hu et al. [48].

We can conclude from Table 2 that tri-GalNAc therapeutics enhance specific and efficient targeting of siRNA to hepatocytes in the liver, while lipid-based siRNA delivery systems offer protection and enhanced cellular uptake for broader cell types, and naked siRNA lacks a delivery system and may have limited stability and cellular uptake efficiency (Figure 4). Thus, the delivery approach is chosen based on the specific target cells, desired efficacy, and therapeutic goals.

### 5.1. Naked siRNA-Based Therapeutics

Naked siRNA-based therapeutics are small interfering RNA molecules administered without a delivery vehicle. Unlike other siRNA delivery approaches that utilize lipid nanoparticles or viral vectors to enhance cellular uptake and stability, naked siRNA therapy involves administering siRNA directly into the body without any complexation or encapsulation. The administration of naked siRNA molecules is typically through injection or inhalation. Although naked siRNA therapy offers simplicity and cost-effectiveness, it faces some challenges that limit its efficacy. The molecules are generally unstable and can be rapidly degraded by nucleases in the bloodstream and extracellular environment, which reduces their bioavailability and restricts their ability to reach target cells.

Additionally, naked siRNA molecules have difficulty crossing the cell membrane to reach the cytoplasm, where they exert their gene-silencing effect. Various strategies have been explored to overcome these challenges and enhance the delivery and stability of naked siRNA. For example, modifications to the siRNA structure, such as using chemically modified nucleotides or incorporating stabilization moieties, can improve direct injection into specific tissues or organs, increasing the chances of siRNA reaching the intended target cells.

#### 5.1.1. QPI-1002

QPI-1002 (teprasiran or I5NP) is a naked siRNA therapeutic developed by Quark Pharmaceuticals. It targets the p53 gene, which plays a role in acute kidney injury (AKI) [128]. Demirjian and co-workers described using intravenous administration of QPI-1002 in a Phase I study. The pharmacokinetics (PK), safety, and tolerability of QPI-1002 in patients undergoing cardiac surgical procedures and at risk of developing AKI were examined [129]. The safety, well-tolerance, and absence of immediate reactions were demonstrated for single intravenous bolus injections of QPI-1002 4 h after cardiopulmonary bypass. The drug quickly eliminated plasma in all four dosing cohorts, with an average residence time between 10 and 13 min. Teprasiran’s efficacy and safety in preventing AKI in high-risk patients admitted for cardiac surgical procedures were evaluated in the Phase 2 trial (NCT02610283). The trial involved a prospective, randomized, multicenter, double-masked, and monitored study. In 41 centers, 360 participants were allocated arbitrarily; 341 dose participants were 73 ± 7.5 years of age, 72% of individuals were men, and the European System for Cardiac Operative Risk Evaluation score was 2.6% [130]. The trial’s main goal was to examine the incidence of AKI in participants who took a single dose (10 mg/kg) of teprasiran compared to placebo. Secondary endpoints such as AKI severity, duration, and serious adverse kidney effects were evaluated in the trial at day 90. The trial revealed that teprasiran administration significantly decreased the incidence, difficulty, and period of early AKI in high-risk individuals undergoing cardiac surgical operations. The incidence rate of AKI in the teprasiran group was 37% compared to the placebo group, with an absolute risk reduction of 12.8%. Teprasiran treatment did not pose any significant safety concerns. QPI-1002 was investigated in the Phase 3 clinical trial (NCT02610296) for its efficacy and safety in preventing delayed graft function (DGF) in recipients of older donor kidney transplants. In cases where the donor is older, delayed graft function is a common complication after kidney transplantation. The trial is intended to be a double-masked, randomized, placebo-monitored study, meaning some participants will receive QPI-1002 while others will receive a placebo. As mentioned above, the primary purpose is to evaluate the degree of deferred graft function in the QPI-1002 cluster compared with the placebo group. The secondary objectives could include assessing kidney function, graft survival, and overall patient outcomes. Notably, the Phase 3 trial results are yet to be published.

#### 5.1.2. SYL040012

SYL040012 (bamosiran) is a naked siRNA-based therapeutic developed by Sylentis (PharmaMar Group). This is the first therapeutic siRNA used for eye treatment [131]. The gene of vascular endothelial growth factor receptor 1 (VEGFR1) is targeted to inhibit angiogenesis in ocular maladies, including age-related macular degeneration (AMD) and diabetic macular edema (DME). The mechanism is to decrease intraocular pressure by reducing the production of intraocular fluid. Bamosiran is administered as an eye drop, which is a novel method of administration for this type of medication. The clinical trials for Bamosiran have been conducted in multiple phases. In healthy participants, the Phase I trial was completed to investigate the safety, tolerability, and PK of bamosiran [132]. The trial was conducted in a randomized, parallel-design, placebo-controlled, double-masked manner. Different doses of bamosiran were evaluated in the Phase 2 study for their tolerability and IOP-lowering effects. Patients with elevated IOP or glaucoma received a daily dose for 14 days [133]. The study concluded that 300 µg/eye/day substantially decreased IOP compared to the placebo and the IOP curve achieved during the assessing period [133]. The substance was well tolerated, and no adverse side effects were recorded. The cost-effectiveness problem will remain unless there is optimization of the siRNA structure and the mode of the nanoparticle’s delivery.

#### 5.1.3. QPI-1007

Quark Pharmaceuticals developed QPI-1007 to treat ocular diseases. Specifically, QPI-1007 targets the gene responsible for producing caspase 2, an enzyme involved in cell death pathways to prevent apoptosis [134]. The drug is being developed for various ocular disorders, namely non-arteritic anterior ischemic optic neuropathy (NAION), primary open-angle glaucoma (POAG), and DME. The expression of caspase 2 is inhibited by QPI-1007, which is thought to play a role in these disorders’ etiology [135]. QPI-1007 is still investigational and has not received regulatory approval for commercial use. The chemical modifications that were added to QPI-1007 siRNA in Ahmed and co-workers’ report on the preclinical of QPI-1007 can be utilized to both protect synthetic siRNA from nuclease degradation and to decrease its off-target and immunostimulatory outcomes [136]. Phase I and II clinical investigations for NAION have been completed for QPI-1007 therapeutic. One notable clinical trial involving QPI-1007 is a Phase 3 evaluation called “QUARK-AMD” (NCT02341560). This study aimed to evaluate QPI-1007’s effectiveness and safety in treating patients with acute NAION.

### 5.2. Lipid-Based siRNA

Lipid-based siRNA delivery systems are a formulation used to deliver small interfering RNA (siRNA) molecules into cells. Lipid-based formulations can improve siRNA’s stability, solubility, and pharmacokinetics, enhancing its delivery to target cells and tissues. Lipid-based siRNA formulations typically comprise several components that work together to form a stable and efficient delivery system. The composition of lipid-based siRNA formulations can vary depending on the specific formulation strategy and desired properties. In the late 1970s, liposomes were used as part of the formulation to encapsulate some NAs, but the systems suffered from poor encapsulation, potency, and tolerability [137]. Permanently charged cationic lipids were also used in earlier lipid formulations, but these conventional cationic lipids exhibit high cytotoxicity due to their strong positive charge.

**Figure 5:**
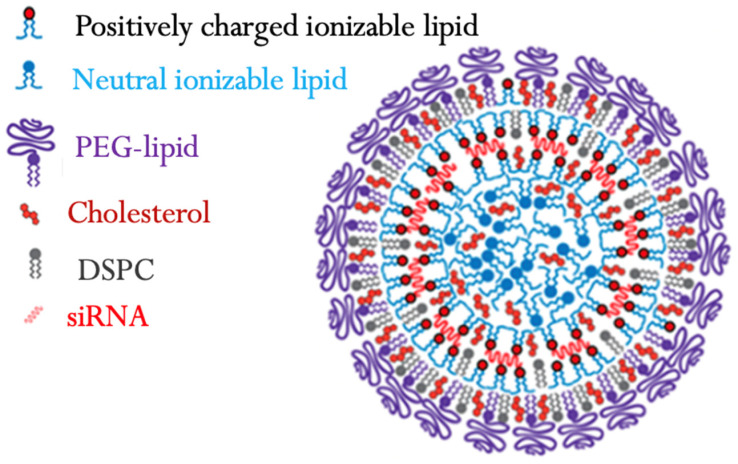
The structural arrangement of lipid nanoparticles (LNPs) containing siRNA. The structural arrangement of LNP-siRNA reveals two separate phases: an oil-like phase, which contains the uncharged ionizable lipids, and an aqueous phase. This organization highlights the separation of components within the nanoparticle system. Reprinted (and adapted) with permission from [139]. Copyright 2018 American Chemical Society.

In contrast, ionizable cationic lipids (Figure 2) show reduced cytotoxicity because they have a variable charge, allowing efficient intracellular delivery while minimizing undesirable interactions with cellular components. These paved the way for the breakthrough of ionizable cationic lipids to develop pharmacologically acceptable lipid formulations. Ionizable cationic lipids have a net positive charge at physiological pH, which allows them to effectively interact with and bind to negatively charged siRNA molecules. However, under the acidic conditions inside the endosomes or lysosomes of cells, the cationic lipids are protonated and become neutral or negatively charged, leading to a charge reversal. This charge reversal facilitates the escape of the lipids and siRNA from the endosomes and promotes their release into the cytoplasm, where they can exert their therapeutic effect. Ionizable cationic lipid formulations provide stability to the siRNA cargo [138]. The lipids protect the siRNA from enzymatic degradation and nuclease attack during circulation, increasing its stability and bioavailability. Examples of commonly used cationic lipids include DOTAP (1,2-dioleoyl-3-trimethylammonium-propane), DOTMA (N-[1-(2,3-dioleoyloxy)propyl]-N,N,N-trimethylammonium methyl sulfate), and DMRIE-DOPE (1,2-dimyristyloxypropyl-3-dimethyl-hydroxyethyl ammonium chloride), among others. Phosphatidylcholine (PC), cholesterol, and phosphatidylethanolamine (PE) are also used in lipid-based siRNA formulations to enhance the siRNA’s stability, cellular uptake, and endosomal escape. These lipids can modify the cationic lipids’ physicochemical properties and improve the delivery system’s overall performance. Polyethylene Glycol (PEG) (Figure 5) is another constituent in lipid-based siRNA formulations to enhance their stability and circulation time in the bloodstream. PEGylation helps avoid recognition by the immune system and reduces the clearance rate, allowing for prolonged systemic circulation. Notably, intravenously administered siRNA LNPs are coated with apolipoproteins that target the LNPs toward the LDL receptor of hepatocytes [138]. The observation that intravenously administered siRNA LNPs are coated with apolipoproteins that target the LDL receptor of hepatocytes epitomizes how researchers have utilized the body’s endogenous mechanisms to enhance drug delivery. This endogenous targeting approach is a significant factor contributing to the success of LNPs in delivering siRNA to the liver.

#### ALN-TTR02

ALN-TTR02 (Patisiran) was designed and developed by Alnylam Pharmaceuticals to treat transthyretin-mediated amyloidosis (ATTR) disorder. It aims to target and impede the transthyretin (TTR) gene, controlling the construction of a protein associated with a rare genetic illness known as hereditary ATTR. Patisiran, marketed under Onpattro, underwent different clinical trials to evaluate its safety and effectiveness. These clinical trials provided valuable data to support the approval of patisiran as a treatment for this rare genetic disease. The Phase 3 clinical trial, the APOLLO study, involved a larger population of patients with hATTR. This trial aimed to demonstrate the clinical benefit of patisiran compared to a placebo [140]. Patients were arbitrarily assigned to either the patisiran or the placebo cluster and received treatment over a specified period. The primary outcome was the improvement in a specific neurologic composite score. Secondary endpoints included quality of life, disease progression, and adverse events. Patisiran at 9 and 18 months lowered the brain natriuretic peptide’s N-terminal prohormone. The ratio of fold-change at 18 months patisiran/placebo was 0.45 (*p*-value < 0.001) [140] with an average follow-up time of 18.7 months. The placebo and patisiran cluster had elevated cardiac hospitalizations and all-cause mortality levels of 18.7 and 10.1/100 patient-years, respectively. This trial showed significant improvements in nerve function and value of life for participants receiving patisiran. Even though patisiran is therapeutic for a rare disease, a few of its shortcomings cannot be overlooked. The route of patisiran administration is through intravenous infusion, which requires healthcare professionals to administer the drug. This can be inconvenient for patients, as it may necessitate frequent visits to a medical setting for treatment. Additionally, the infusion process can be time-consuming, lasting several hours, which may further inconvenience patients and limit their ability to engage in regular daily activities.

### 5.3. GalNAc-siRNA Conjugates 

GalNAc-siRNA conjugates are a therapeutic approach combining siRNA molecules with *N*-acetylgalactosamine (GalNAc) molecules. This conjugation allows for targeted delivery of siRNA to hepatocytes, which are liver cells that play a crucial role in many diseases. GalNAc-siRNA conjugates work by taking advantage of the natural uptake mechanisms of hepatocytes. Tri-GalNAc is a ligand that binds to the asialoglycoprotein receptor (ASGPR) on the surface of hepatocytes. ASGPR recognizes GalNAc, leading to the internalization of the GalNAc-siRNA conjugate into the cell. Once inside the hepatocyte, the siRNA portion of the conjugate can target and bind to specific mRNA molecules, leading to their degradation and subsequent reduction in the expression of target genes. This gene-silencing effect can therapeutically benefit liver-related diseases, including viral infections, metabolic disorders, and liver cancers [9,141]. Using GalNAc-siRNA conjugates has demonstrated promising results in preclinical and clinical studies. It is important to note that monomeric GalNAc exhibits low, insufficient affinity.

GalNAc-siRNA conjugates are typically administered via subcutaneous injection [142]. This route of administration involves injecting the compound into the subcutaneous tissue, as opposed to intravenous administration. Subcutaneous injection facilitates the absorption of the conjugate into the systemic circulation, subsequently enabling efficient hepatic targeting [143]. This mode of administration contributes to the reduction in siRNA exposure to non-target tissues, thereby mitigating the potential for off-target effects. Subcutaneous injections generally offer greater ease of administration compared to intravenous infusions, thus enhancing patient compliance and convenience.

#### 5.3.1. Givosiran

Alnylam Pharmaceuticals has developed givosiran to treat acute hepatic porphyria (AHP), a rare genetic disorder. It targets and lessens the production of a specific enzyme in the liver called aminolevulinic acid synthase 1 (ALAS1), thereby decreasing the buildup of lethal substances that cause disease symptoms. Givosiran is delivered subcutaneously and has proved effective in reducing porphyria attacks. In a Phase 1/2 clinical trial, givosiran was administered subcutaneously to patients with hereditary coproporphyria (HCP) and acute intermittent porphyria (AIP). The study demonstrated that givosiran substantially decreased the levels of ALAS1 messenger RNA and urinary excretion of ALA, a biomarker of AHP activity [144,145]. The drug also reduced porphyria, decreased hemin usage, and enhanced quality of life [146]. The pivotal Phase 3 study, known as ENVISION, evaluated the safety and effectiveness of givosiran in participants with AHP. The ENVISION trial, a randomized, double-blind, placebo-controlled study, was investigated in participants with AHP who experienced persistent attacks [147]. During the trial, participants were arbitrarily allotted to take either givosiran or a placebo. The treatment was administered subcutaneously (under the skin) once every month. The trial’s primary result was the decrease in the annualized rate of porphyria incidents in the givosiran-treated cluster compared to the placebo cluster. The study also showed reduced ALA levels and a favorable safety profile. Based on the favorable outcome from the Phase 1/2 and Phase 3 investigations, givosiran has received regulatory approvals in the United States (brand name—Givlaari) and the European Union as a medication to treat AHP.

#### 5.3.2. Vutrisiran

Vutrisiran was an investigational therapeutic agent developed by Alnylam Pharmaceuticals to treat hATTR. Vutrisiran is designed and developed to attack and silence the production of abnormal TTR protein, thus potentially reducing or blocking the progression of the disease disorder. In preclinical studies, vutrisiran reduced serum TTRs by 55% and 96%, respectively, with non-human primates administered as a single SC dose at 0.3 and 1 mg/kg. TTR levels were reduced by 96% at 1 mg/kg and 3 mg/kg per month, compared to baseline, in the same study [148]. The Phase 1 trial of vutrisiran investigated the drug’s safety, tolerability, PK, and pharmacodynamics (PD) in healthy and unhealthy individuals with hATTR amyloidosis [148]. The study proved that vutrisiran demonstrated a dose-dependent decrease in TTR amounts, which is the target of the drug. A single 25 mg SC dose of vutrisiran led to a maximal TTR decrease of 80% in healthy subjects, and this effect lasted for nearly 90 days. This is comparable to the TTR decrease with patisiran q3w (83–84%) in the APOLLO Phase 3 investigation, where the magnitude of TTR decrease was linked to enhancement in polyneuropathy and a broad spectrum of clinical symptoms. The 25 mg three times a year regimen of vutrisiran is expected to reduce TTR by 88–90% consistently. The vutrisiran regimen of 25 mg every three months is projected to result in a therapeutic use similar to the result examined in the APOLLO investigation when taken as a whole. This suggests that vutrisiran effectively inhibits the production of TTR protein, which is a key driver of hATTR amyloidosis. Regarding safety, vutrisiran was highly effective in healthy volunteers and patients with hATTR amyloidosis. The most challenging events in the investigation were mild to moderate, typically infusion-related reactions, and there were no serious safety concerns. This indicates a favorable safety profile for vutrisiran. Based on these positive Phase 1 results, further clinical development of vutrisiran has been conducted in Phase 2 and Phase 3 trials to assess its effectiveness and safety in larger patient populations.

Vutrisiran’s efficacy and safety were evaluated in individuals with hATTR amyloidosis and polyneuropathy in the HELIOS-A trial in a Phase 3 study [149]. The investigation showed that vutrisiran extensively decreased serum TTR levels compared to placebo and demonstrated a favorable safety profile. Vutrisiran’s effectiveness and safety were comparable in HELIOS-A with those of a placebo group (APOLLO). For 18 months, the participants were randomized to take either subcutaneous vutrisiran 25 mg every three months (Q3M) at a 3:1 ratio or to take 0.3 mg/kg of intravenous patisiran every three weeks (Q3W). 164 patients have been enrolled in HELIOS-A (122 vutrisiran users, 42 patisiran users serving as the control group, and 77 external placebo users). The results showed that the tested drug (Q3M) was not inferior to patisiran (Q3W) in terms of TTR decrease. In line with the typical course of ATTRv amyloidosis, most side effects were mild to moderate in severity. Both drug-related fatalities and discontinuations were absent. The ongoing HELIOS-B trial is a comprehensive Phase 3 study examining the effectiveness and safety of vutrisiran in individuals with hATTR amyloidosis. The primary purpose is to investigate the efficacy of vutrisiran on neuropathy progression and other clinical endpoints. Enrollment in the trial is underway, and the year the study will be completed is December 2026. The Phase 3 study of vutrisiran would examine its continuing safety, admissibility, and efficacy in participants with ATTR amyloidosis. This study would typically involve many participants (655), randomized to take vutrisiran or a placebo, and monitored for an extended duration to assess various endpoints such as disease progression, reduction in TTR protein, and improvement in clinical symptoms.

#### 5.3.3. Cemdisiran

Cemdisiran (developed by Alnylam Pharmaceuticals) targets and obstructs the assembly of a specific protein known as complement component 5 (C5), which plays a role in the complement system, an immune system component. By inhibiting C5 production, cemdisiran aims to potentially treat diseases involving the complement system’s dysregulation. Cemdisiran is currently in clinical trials to treat PNH, a blood disorder that is rare and life-threatening. The results from Phase 1/2 indicate that cemdisiran was highly effective in healthy and unhealthy participants with PNH [150]. Most undesirable events were slight to moderate in severity, with no significant. Cemdisiran achieved a robust and sustained reduction in C5 levels and inhibition of complement activities. Additionally, more than an 80% decrease in complement pathway activity for over 20 days was observed using a single dose of 600 mg. Despite eculizumab’s poor response, the C5 level showed a similar reduction after receiving 600 mg of cemdisiran in a healthy Japanese volunteer who was heterozygous for an Arg885His polymorphism in the C5 gene [150]. This suggests that cemdisiran’s mechanism of silencing C5 mRNA is independent of this polymorphism. In eculizumab-naive PNH patients, the hemolysis was not fully controlled by cemdisiran monotherapy. However, in patients already on eculizumab, cemdisiran allowed for reduced eculizumab dosing while maintaining LDH normalization and complement inhibition for up to 6 months after cemdisiran dosing ended. The prolonged C5 suppression from cemdisiran supports less frequent dosing, potentially every month. This could be advantageous compared to intravenous administration. Cemdisiran could be the first RNAi therapeutic for complement-mediated diseases and represent a novel treatment alternative for patients with PNH and other complement-driven disorders. Phase 2b and 3 trials are being investigated to assess the drug’s safety, efficacy, and optimal dosing for PNH patients.

#### 5.3.4. Nedosiran

Dicerna Pharmaceuticals is investigating DCR-PHXC therapeutic agents to treat primary hyperoxaluria type 1 (PH1) and PH2, rare genetic disorders characterized by excessive urinary oxalate (Uox) accumulation in the body. DCR-PHXC works by targeting and inhibiting the production of glycolate oxidase and lactate dehydrogenase, enzymes responsible for the overproduction of oxalate in PH1 and PH2 patients. In the Phase 1 trial (PHYOX1), the molecule was monitored for its safety therapeutic effect in primary hyperoxaluria (PH) [151]. The PK of nedosiran was linear and dose-proportional across the tested dose range. Nedosiran’s safety profile was satisfactory in healthy volunteers and PH1 or PH2 patients. Administration site adverse reactions were the most frequent treatment-related side effects. When the investigating drug was used therapeutically, there was a tendency in 24-h Uox excretion that decreased with dose while increasing in nedosiran systemic exposure. According to nonclinical evidence, Nedosiran enters hepatocytes, suppressing LDHA mRNA and, consequently, LDH enzymatic activity [151,152]. To treat phosphate hyperphosphatemia in people with PH, PHYOX1 results justify using a constant dose of 160 mg of nedosiran monthly. This dosage should minimize rises in oxalate levels between amounts that can cause acute renal damage. The PHYOX2 Phase 2 clinical trial evaluated the effectiveness and safety of nedosiran in patients with PH1 or PH2 [153]. In a double-masked, placebo-controlled trial, the evaluation involved 35 individuals with PH1 (n = 29) and PH2 (n = 6). For six months (NCT03847909), subcutaneous nedosiran or placebo in a ratio of 2:1 was administered to participants once a month. The study’s primary outcome was the decrease in Uox excretion, measured by the area under the curve (AUC) between days 90 and 180. The findings have shown that nedosiran substantially boosted the AUC percent reduction in Uox excretion when compared to placebo (least squares mean [SE], +3507 [788] vs. −1664 [1190], respectively). Additionally, it was observed that more patients subjected to nedosiran experienced normal or nearly normal Uox excretion on day 90. The PH1 subgroup experienced the most substantial impact of this effect (64.7% versus 0; *p* 0.001). Likewise, those with PH1 who received nedosiran demonstrated significantly lower plasma oxalate levels than placebo (*p* = 0.017). The safety profile of nedosiran was generally favorable, with injection-site responses as a common adverse effect. In conclusion, the PHYOX2 trial demonstrated that nedosiran treatment led to clinically meaningful lessening of Uox excretion in patients with PH1, which is the crucial reason for kidney impairment in PH. The study also suggested a potential benefit of nedosiran in PH2, although the effect was not as consistent as in PH1. Further studies, including more extensive Phase 2 and 3 trials, are under investigation to evaluate the optimal dosing, effectiveness, and safety of nedosiran in this patient population [154].

#### 5.3.5. Lumasiran

Lumasiran is an RNAi therapeutic designed by Alnylam Pharmaceuticals to treat PH type 1. It works by silencing the L-glyceric acid oxidase (GAO) gene, which is responsible for the overproduction of oxalate in PH1 patients. By decreasing the assembly of oxalate, lumasiran helps prevent the accumulation of oxalate crystals and the resulting damage to the kidneys and other organs. Lumasiran has undergone clinical trials to investigate its safety, efficacy, and PK in individuals suffering from PH1. Lumasiran’s clinical trials have demonstrated its effectiveness in reducing Uox levels and improving kidney function in individuals with PH1 [155]. The primary outcome from baseline to 6 months was the percent alteration in 24-h Uox excretion. According to the findings, patients who received lumasiran experienced a 65% drop in urinary oxalate levels compared to the placebo group’s 11% increase. The urinary oxalate reduction was at least 50% in 84% of patients who received lumasiran, compared to 0% in the placebo group. Additionally, secondary endpoints included reductions in the number and size of kidney stones and improvements in the projected glomerular filtration rate (eGFR). Patients who finished the ILLUMINATE-A trial were included in the ILLUMINATE-B open-label extended trial to evaluate the lasting safety and effectiveness of lumasiran [156]. The findings showed a continued decrement in urinary oxalate levels with sustained efficacy over a 30-month treatment period. No new safety concerns were identified, and the adverse effects, such as injection site reactions, were minor to moderate. Lumasiran has consistently demonstrated reductions in urinary oxalate levels and improved kidney function and maintains an appropriate safety report in clinical trials. Meanwhile, the Phase 3 ILLUMINATE-C trial evaluated the efficacy, safety, PK, and PD of lumasiran in individuals with PH1 and complex kidney infection [157]. It was proven that lumasiran reduced oxalate levels in participants with PH1 and relatively conserved kidney function. Plasma oxalate levels decreased significantly by 33.3% to 42.4% in the two patient cohorts during the 6-month study period. Urinary oxalate levels and other oxalate measures also improved with lumasiran treatment. No patients withdrew therapy due to side effects. According to the findings, lumasiran can be advantageous in decreasing oxalate levels and is safe to utilize in individuals with PH1 and later stages of kidney disease, including those on hemodialysis. Lumasiran got FDA approval in late 2020 under the brand name Oxlumo to treat PH1 in adults and pediatric individuals aged six years and older. It is administered via subcutaneous injection every three months.

#### 5.3.6. ARO-APOC3

ARO-APOC3 is another siRNA investigational drug designed by Arrowhead Pharmaceuticals to treat patients with familial chylomicronemia syndrome (FCS). ARO-APOC3 is intended to silence the APOC3 gene by reducing APOC3 protein production, lowering triglyceride levels in the bloodstream. FCS is a rare genetic syndrome characterized by severely elevated triglyceride levels and a buildup of chylomicrons in the blood. It can cause dangerous pancreatitis as well as other health issues. There are currently no approved drugs specifically for FCS. When ARO-APOC3 was assessed in APOC3 transgenic mice (human), liver mRNA decreased in a dose-dependent manner, and serum human APOC3 protein (maximum 91%) as well as decreases in TGs and LDL-C were noted [158]. Reduced TGs, LDL-C, and serum APOC3 (up to 80%) were seen in a dyslipidemic NHP model, and the magnitudes of the reductions were associated with the NHPs’ level of dyslipidemia. In early-stage clinical trials, ARO-APOC3 has shown potential in reducing triglyceride levels in FCS patients [159,160,161]. Arrowhead is currently conducting a Phase 2b trial to further examine the safety and effectiveness of ARO-APOC3 in FCS. ARO-APOC3 could be the first drug specifically indicated for treating FCS if approved. Arrowhead hopes to file for regulatory approval in 2024.

#### 5.3.7. SLN360

SLN360 is a lipoprotein (a)-related cardiovascular risk-reducing siRNA that targets liver-targeting N-acetyl galactosamine (GalNAc) [162]. Following subcutaneous administration of SLN360, serum Lp(a) levels are reduced in cynomolgus monkeys [162]. In the APOLLO Phase 1 clinical trial, 32 adults from five hospitals in the United States, UK, and the Netherlands were registered to evaluate SLN360 [163]. A median level of 224 nmol/L was found in all participants at screening (75 nmol/L is considered normal). In this study, subjects were randomly administered 30 mg, 100 mg, 300 mg, or 600 mg of SLN360 subcutaneously. The patients were closely observed for the first 24 h, and then, for 150 days, they were evaluated periodically. Injection site redness was the most common side effect, but no serious safety concerns were reported. A median decrement in Lp(a) levels was observed in participants (96% and 98%) taking 300 mg or 600 mg of SLN360; further reductions were observed in participants, and ~71% and 81% from baseline continued at 150 days. Lp(a) levels did not change in those receiving placebos. For a more thorough assessment of the duration of action, the monitoring cycle has been extended to 365 days. Apolipoprotein B (ApoB) is an apolipoprotein that may cause cardiovascular events, but SLN360 also reduces LDL cholesterol and ApoB. The most effective doses of SLN360 lowered LDL cholesterol and ApoB by approximately 25%. There is an unmet need for lowering Lp(a) in cardiovascular diseases (CDs), which can be addressed by SLN360 [162].

#### 5.3.8. RBD1016

RBD-1016 is an investigational therapeutic agent developed by Suzhou Ribo Life Science. It is a GalNAc-conjugated siRNA therapy based on Ribo’s registered liver-targeting delivery technology RIBO-GalSTAR, aiming at the conserved region of the X gene hepatitis B [164]. In the Phase 1 trial, participants were split into a single-dose and a multiple-dose cluster and were administered RBD1016 subcutaneously. From the Phase 1 clinical trial, RBD1016 significantly decreased the amounts of hepatitis B surface antigen (HBsAg) over a 24-week period compared to placebo in individuals with chronic HBV illness. A rapid and lasting decrease in serum HBsAg was observed with a single dose of RBD1016 in a dose-dependent manner. Multiple doses of RBD1016 showed a significant reduction in HBsAg. Maintaining HBsAg suppression for this duration is an imperative proof-of-concept for the potential of the siRNA approach. Preliminary safety data suggested that RBD1016 was safe and well tolerated. This sustained decrease in HBsAg is a positive indicator of the potential efficacy of RBD1016 in treating chronic hepatitis B [165].

#### 5.3.9. DCR-A1AT

Belcesiran is being designed as a potential therapeutic to treat alpha-1 antitrypsin (A1AT) deficiency, a genetic disorder that can lead to tissue diseases (lung and liver). A1AT deficiency is characterized by a lack of the A1AT protein, which usually protects the lungs and liver from damage caused by enzymes released by white blood cells; however, there is still a therapeutic gap for liver illness linked to A1AT deficiency. A single subcutaneous dose of belcesiran was tested in a Phase 1 study on 30 adult healthy volunteers to determine its safety, tolerability, PK, and PD compared to a placebo. Belcesiran decreased serum AAT in humans at dosages as high as 6 mg/kg, with the most significant decreases happening about eight weeks after the treatment [166], the 12 mg/kg and 6 mg/kg cohorts achieved comparable reductions. When doses greater than 0.1 mg/kg were administered, mean serum AAT reductions from baseline were 49.7% (1.0 mg/kg), 67% (3.0 mg/kg), 77% (6.0 mg/kg), and 78% (12.0 mg/kg). The highest reduction occurred almost eight weeks after dose administration. About 90% of serum AAT was reduced in two participants after receiving belcesiran 6 mg/kg. Regarding treatment-related adverse events (TEAEs), none have been reported as serious or severe. To probe an alternative RNAi drug, belcesiran, the ESTRELLA Phase 2 clinical trial is in an active phase and no longer recruiting patients with AATD-associated liver disease transplantation (NCT04764448). Belcesiran’s effectiveness, tolerability, PK, and PD in individuals with A1AT deficiency-related liver ailment are being assessed in the Phase 2 ESTRELLA trial, a randomized, multidose, double-masked, placebo-controlled investigation. Dicerna’s clinical development program for belcesiran involves conducting rigorous research to evaluate its safety and efficacy. These trials are designed to gather data on the drug’s effectiveness in treating A1AT deficiency and its potential benefits for patients. Therefore, if the clinical study is successful, belcesiran could fill the therapeutic gap for the cohort of individuals with AATD-mediated liver disease who lack treatment choices other than liver [167].

#### 5.3.10. Olpasiran

Olpasiran (AMG 890) is being studied for its potential to reduce Lp(a) levels in patients with CD. The mechanism of action of olpasiran involves using small RNA molecules that can bind to the messenger RNA (mRNA) molecules that code for the assembly of Lp(a). By binding to the mRNA, olpasiran prevents its translation into protein, effectively reducing the accumulation of Lp(a) in the liver. Olpasiran has shown promising results in early clinical trials, demonstrating significant Lp(a) level reductions without major safety concerns. These positive findings have led to further investigations, including ongoing Phase III clinical trials, to investigate the safety and long-term use of olpasiran on CD. Olpasiran could achieve a continuous reduction of >80% in plasma Lp(a) based on data amassed from in vivo studies using transgenic mice and cynomolgus monkeys [168]. The evaluation of the Phase 1 clinical investigation of olpasiran showed promising findings. The study evaluated the PD (PD), PK (PK), and effectiveness of a single subcutaneous (SC) injection of olpasiran in healthy Japanese and non-Japanese individuals [169]. While non-Japanese individuals received 75 mg of olpasiran, Japanese individuals were given a single dose of 3, 9, or 225 mg. According to the findings, olpasiran was rapidly absorbed, with an average time to highest concentration (tmax) varying from 3.0 to 9.0 h across all dose levels. The Japanese cohorts showed a dose-proportional increase in the maximum concentration (Cmax) and AUC values. The mean Cmax values were between 144 and 242 ng/mL, and the average AUC values were between 2620 and 3550 h.ng/L. Olpasiran showed a decrease in Lp(a) levels that was dose-dependent in terms of PD.

Across all dose cohorts, the maximum mean percentage reduction from baseline was between 56.0% and 99.0%. Lp(a) decreased as early as day four and persisted throughout the study, with a mean percentage reduction of 68% at the 75 mg dose. Olpasiran was tolerated well, with no reports of severe or fatal adverse events. These findings support the development of olpasiran as a probable therapeutic option for reducing Lp(a) levels. To examine the effectiveness and safety of olpasiran in lowering lipoprotein(a) concentrations in individuals with established ASCVD and high Lp(a) levels (NCT04270760), the Olpasiran trials, also known as the OCEAN(a)-DOSE trials of cardiovascular events and Lp(a) reduction-DOSE finding study (OCEAN(a)-DOSE), were designed [170,171]. The trials aimed to examine the dose-dependent effects of olpasiran on Lp(a) reduction and determine the optimal dosing regimen for further evaluation in a cardiovascular outcomes trial. The OCEAN(a)-DOSE trials were designed as a dose-finding study with a multicenter, randomized, double-blind, placebo-controlled design. 281 individuals with proven ASCVD and elevated Lp(a) levels (>150 nmol/L) were enrolled and given one of four active subcutaneous doses of olpasiran randomly (10 mg/12 weeks, 75 mg/12 weeks, 225 mg/12 weeks, or 225 mg/24 weeks) or matched placebo. The main aim of the trials was to investigate the effects of olpasiran administered every 12 weeks compared to placebo on the percentage change in Lp(a) from baseline at 36 weeks. Secondary objectives included assessing the safety and tolerability of olpasiran and exploring its effects on other lipid parameters. The trial results showed that olpasiran therapy administered every 12 weeks successfully decreased the Lp(a) concentration in a dose-dependent manner; a 10 mg dose caused a placebo-adjusted mean percent change of 70.5%. At the same time, the 75 mg dose resulted in −97.4%, −101.1% with the 225 mg dose, and −100.5% with the 225 mg dose administered every 24 weeks.

#### 5.3.11. Inclisiran

Inclisiran targets a specific gene involved in producing LDL cholesterol called PCSK9. By inhibiting PCSK9, inclisiran eliminates LDL cholesterol from the bloodstream, lowering LDL cholesterol levels [172]. Tri-GalNAc is conjugated with inclisiran on the sense strand to facilitate hepatocyte uptake. The safety and effectiveness of inclisiran have been shown in numerous clinical trials, such as the ORION scheme. Inclisiran was given as a subcutaneous injection to participants with a high risk of CD (CD) in Phase 2 clinical trial (ORION 1) [173]. The individuals were given either a single dosage of 200, 300, or 500 mg, a double dose of control (given on days 1 and 90), or a dose of 100, 200, or 300 mg of inclisiran at random. At 180 days, inclisiran treatment significantly lowered LDL cholesterol levels compared to placebo in patients with a high risk of CD. It elevated LDL cholesterol levels despite taking the highest dose of statin medication. Levels of PCSK9 were likewise dramatically decreased in patients who took inclisiran at 180 days, which is consistent with this outcome and the drug’s intended use. The two-dose 300 mg regimen of inclisiran resulted in the most significant reduction (52.6%) in LDL cholesterol levels; this decrease is comparable to that achieved with PCSK9-targeting monoclonal antibodies [174,175]. Other lipid measures changed in patients receiving inclisiran and were likewise generally consistent. It was concluded in the ORION-1 clinical trial after a one-year follow-up that a single dose of inclisiran can result in a 36.6% decrease in LDL-C over a year and a 50% decrease in LDL-C for at least six months after taking two doses of 300 mg on day 1 and day 90 [176]. By giving inclisiran to patients on day 1, day 90, and twice a year after, doctors can help patients maintain a decrease in their LDL-C levels.

In the ORION-2 study, four participants were treated, and one participant enrolled withdrew consent before the treatment [177]. The rationale of this study was to confirm the validity of a long-term phase 3 trial (ORION-5). Patients were administered inclisiran on the first day and day 90. However, the fourth patient was only administered inclisiran on day one because their PCSK9 levels were still suppressed by >70%. All four individuals achieved a robust and long-lasting reduction in PCSK9, with high-intensity statins and an ezetimibe background that ranged from 48.7 to 83.6% at day 90 and 40.2 to 80.5% at day 180, according to the outcome results. A decrease in LDL-C was observed between 11.7–33.1% at day 90 and 17.5–37.0% at day 180 in the second to fourth patients. However, no reduction in LDL-C levels was observed in the first patient. The durability of cutbacks in other atherogenic lipids and lipoproteins was confirmed by the ORION-3 trial [178]. The inclisiran-only arm received 290 of the 370 patients assigned to the drug, and 92 out of 127 individuals assigned to placebo entered the switching arm in the ORION-3 extension study. The LDL cholesterol decreased by 47.5%/day 210 in the inclisiran-only arm and was maintained for over 1440 days. The average decrease in LDL-C cholesterol over four years was 44.2%, with declines in PCSK9 from 62.2% to 77.8%, respectively. Also, 39 (14%) of 284 individuals in the inclisiran group and 12 (14%) of 87 participants in the switching arm reported adverse events at the injection site. There were 1% (3 of 284) significant side effects in the inclisiran group and 1% (one of 87) in the switching arm.

The ORION-4 (NCT03705234) and VICTORION-2 Prevent (NCT05030428) trials of inclisiran are ongoing. Despite statin therapy, ~15,000 participants with ASCVD and elevated LDL-C will be enrolled in each of these trials [179]. The median follow-up is expected to be approximately 5 years, with an accumulation of ~1600 to 1700 adjudicated events.

The ORION-6 (Phase 1) study investigated the PK and PD of inclisiran in patients with hepatic impairment [180]. Inclisiran is a new therapeutic agent used to treat hypercholesterolemia. The study included individuals with varying degrees of hepatic impairment, ranging from mild to severe. The PK of inclisiran was evaluated by measuring its concentration in the blood over time. The PD of inclisiran was assessed by monitoring its effect on low-LDL-C levels. The findings from the ORION-6 investigation revealed that the PK of inclisiran was not substantially affected by hepatic impairment. This suggests that dose adjustments may not be necessary for individuals with hepatic impairment when using inclisiran. Furthermore, inclisiran effectively lowered LDL-C levels in individuals with hepatic impairment, similar to patients without hepatic impairment. This indicates that inclisiran remains efficacious in individuals with hepatic impairment, providing a potential treatment option for individuals with hypercholesterolemia and compromised liver function.

The ORION-8 study was a meta-analysis of randomized trials of individuals participating in the phase III ORION-3, ORION-9, ORION-10, and ORION-11 [181]. These studies included a mix of high-risk individuals with ASCVD or risk equivalents, and the average duration of treatment was 2.6 years. Most participants were taking a statin, including 68.5% on high-intensity therapy, and 16.6% were taking ezetimibe. Overall, 78.4% of patients achieved the desired LDL-cholesterol target at the end-of-study visit, with LDL levels reduced by 49.4% from baseline. Among the ASCVD patients, LDL levels were reduced by 51.0% from baseline, and 79.4% achieved an LDL level lower than 70 mg/dL. For those with ASCVD risk equivalents, inclisiran lowered LDL levels by 42.4% and got 74.3% to the goal of less than 100 mg/dL. Adverse events associated with the study drug appeared in 9.1% of patients, but just 2.4% had side effects that led to treatment stoppage.

Further, Phase 3 clinical trials of inclisiran, such as ORION-9, 10, and 11, were performed. ORION-9 included 482 individuals with HeFH or ASCVD who have been administered statin medication. The study demonstrated that inclisiran diminished LDL-C levels by an average of 51% at day 510. ORION-10 included 1561 individuals with ASCVD or ASCVD-risk equivalents who required additional LDL-C reduction despite the maximum tolerated statin treatment and other lipid-lowering therapies [182]. The study revealed that inclisiran diminished LDL-C levels by an average of ~52% at day 510. Also, ORION-11 focused on individuals with HeFH. It included 1617 patients who could not attain LDL-C control with maximally tolerated statin and other lipid-lowering therapies [182]. The trial demonstrated a reduced LDL-C level of ~50% with inclisiran at day 540. These clinical trials showed that inclisiran administered subcutaneously twice a year efficiently lowered LDL-C levels in individuals with elevated cholesterol, with sustained reductions over an extended period. Table 3 describes other examples of tri-GalNAc conjugate therapeutic agents in clinical trials.

## 6. Successes, Failures, and Lessons Learned So Far from the Development of siRNAs

**Figure 6:**
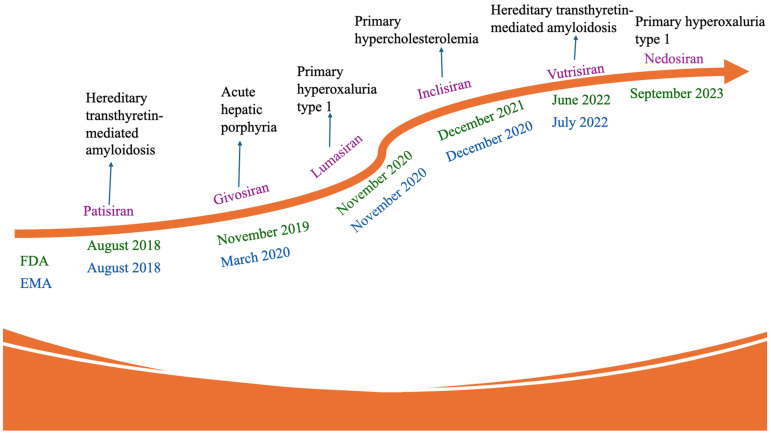
FDA- and EMA-approved siRNA drugs [184].

In truth, siRNA technology has two sides of a coin; it offers excellent potential in terms of targeted gene silencing and therapeutic applications, but on the other, it poses several challenges and limitations that need to be addressed carefully. It can be concluded from the clinical studies conducted on some of the siRNA therapeutics in the past years that it is safe and effective for liver gene knockdown [118]. The success of patisiran in the treatment of hTTR with polyneuropathy, coupled with the development of inclisiran targeting a prevalent patient population rather than a rare disease, underscores the therapeutic potential of siRNA technology. At the same time, failures like bevasiranib and QPI-1007 underscore the challenges and limitations that need to be addressed to improve the clinical success of siRNA therapies. QPI-1007 therapeutic targeting caspase 2 for the treatment of optic nerve injury failed to meet its primary endpoint in clinical trials and did not demonstrate a significant improvement compared to the placebo. Bevasiranib targeting vascular endothelial growth factor (VEGF) for treating age-related macular degeneration (AMD) failed to show significant efficacy in clinical trials. It was also accompanied by severe side effects, thus ultimately discontinued. Our understanding of the critical interactions between nanoparticles and biological systems will deepen as we learn lessons from the clinical transformation of siRNA therapy. Despite the relatively limited number of currently approved siRNA-based therapeutics (six as of the referenced Figure 6), siRNA technology demonstrates significant potential for maintaining a substantial role in the treatment of rare diseases. This implies that even with a small number of approved drugs so far, the potential of siRNA for addressing rare diseases remains high, and further development and approvals are anticipated. Meanwhile, due to recent capital ventures in biotechnology companies, siRNA technology is becoming increasingly attractive for treating other diseases, such as ocular, infectious, and orphan diseases, as well as for modified immunotherapy, thus opening up new possibilities for siRNA technology.

Currently, most siRNA drugs are administered through subcutaneous injections. However, oral delivery would offer several advantages, such as improved patient convenience, increased compliance, and potentially broader patient populations. Efforts are underway to develop effective oral delivery systems for siRNA [185]. One approach involves using delivery systems or carriers that can protect the siRNA molecules from degradation in the gastrointestinal tract and facilitate their absorption into the bloodstream. Various strategies, including nanoparticle-based formulations and chemical alterations, are being explored to enhance the stability and bioavailability of siRNA in the oral route. While progress has been made in this field, it is important to note that oral siRNA delivery is still an active area of research and development. It may take some time before oral siRNA therapeutics become widely available. Nonetheless, the potential benefits of this delivery method make it an exciting prospect for the future of siRNA therapies.

The use of chemical modifications may result in decreased dosage requirements (from milligrams to micrograms) and a longer biological half-life (from minutes to months) [186]. It is imperative to weigh the potential loss of efficacy and toxicity against extensive chemical modification. Carefully considering benefits and risks is also necessary when choosing carrier molecules and excipients [186]. Since RNAi-based drugs are convenient, rapidly adequate, and require less frequent dosing, they could replace specific small molecules and biologics. Other advantages are as follows:

***Broad-spectrum activity***

siRNAs can be designed to target conserved regions of viral genomes, allowing for a broad-spectrum antiviral effect against multiple strains or species of viruses. This versatility is especially beneficial in the treatment of rapidly evolving viruses. For example, there is a need for rapid development of antiviral drugs for coronavirus and its variants. Likewise, siRNA drugs can be used in treating human immunodeficiency virus (HIV), human papillomavirus (HPV), norovirus, monkeypox, influenza virus (INFV), hepatitis B virus (HBV), and West Nile virus (WNV).

***Potent antiviral activity***

siRNAs can effectively silence almost any viral gene expression, inhibiting viral replication and spread. This means that siRNA-based antiviral therapy can efficiently fight infectious diseases by interfering with posttranscriptional gene silencing [187]. This potent mechanism of action can result in a rapid and significant reduction in viral load compared to conventional antiviral drugs [40].

***Rapid therapeutic response***

siRNAs can induce a fast response to new strains of viruses by minimal changes in siRNA sequences within a short period after administration, leading to a quick therapeutic reaction [188]. This rapid action can help control viral replication and limit the progression of the infection. Additionally, siRNA synthesizing and manufacturing on a large scale is relatively easy, and identifying highly selected and inhibitory sequences is much faster than discovering new chemicals.

***Isoform-specific modulation***

Isoforms are different versions of a gene that arise from alternative splicing or other mechanisms, resulting in proteins with slightly different functions or properties. Designing siRNAs complementary to the unique sequence of that isoform while avoiding sequences shared with other isoforms can be explored to target a specific gene isoform. The designed isoform-specific siRNAs should selectively target the mRNA of the desired isoform without affecting other isoforms or closely related genes. This is crucial to avoid unintended off-target effects and ensure that the intended isoform is modulated. siRNA exerts its effect by targeting the mRNA of the specific isoform, leading to its degradation and decreased protein expression. This approach directly interferes with the expression of the target isoform at the mRNA level. In contrast, conventional drugs typically target proteins or pathways involved in a particular disease process. They can affect multiple isoforms of a gene or target downstream signaling pathways rather than directly modulating the expression of a specific isoform. The inherent nucleotide-level specificity of siRNA can be harnessed to develop therapeutics that target isoform-specific exons in genes exhibiting differential splicing patterns in different cancer cells and neurodegenerative disorders [189,190]; this may be difficult using traditional approaches.

***Combinatorial Approaches***

Combining siRNA and traditional drugs is a promising strategy that aims to leverage the benefits of both modalities to enhance therapeutic outcomes. Combining siRNA with conventional medications can lead to synergistic effects, where the two modalities work together to target different aspects of disease pathogenesis. The synergistic effects observed from combining siRNA with one or more traditional drugs to target various aspects of disease pathogenesis can result in enhanced efficacy compared to using either treatment alone. Combining these modalities makes it possible to disrupt multiple disease-related processes simultaneously, leading to a more comprehensive therapeutic response and reduction in the likelihood of resistance development, thus improving treatment outcomes. Combining Alnylam’s cemdisiran with Regeneron’s pozelimab for treating paroxysmal nocturnal hemoglobinuria (PNH) is an investigational therapy in clinical trials. Pozelimab is a monoclonal antibody that targets factor C5, a key enzyme in the complement system for treating paroxysmal nocturnal hemoglobinuria. At the same time, cemdisiran is a siRNA that targets the complement component C5, also part of the complement system. Targeting different components of the complement cascade, pozelimab, and cemdisiran could synergistically modulate the inflammatory response and complement activation more effectively [191]. The dual targeting of pozelimab and cemdisiran could offer more significant therapeutic benefits than targeting either component alone, leading to improved disease outcomes. Thus, pozelimab and cemdisiran could be explored for conditions where dysregulation of the complement system plays a significant role, such as atypical hemolytic uremic syndrome (aHUS), PNH, age-related macular degeneration (AMD), and other autoimmune or inflammatory diseases.

## 7. Conclusions

Since their discovery, RNA interference (RNAi) technologies have undoubtedly embarked on a remarkable journey. From its humble beginnings in the nematode *Caenorhabditis elegans* (*C. elegan*) to its widespread application in research labs and potential clinical settings, RNAi has proven to be a powerful tool with far-reaching implications. Through continuous innovation and technological refinement, RNAi has revolutionized our ability to study gene function and unlocked new therapeutic intervention possibilities. RNA interference technologies have a bright future, helping researchers unravel gene regulation complexity and exploit siRNA’s therapeutic potential, offering new opportunities for tailored, effective therapies.

This review has explored the multifaceted landscape of siRNA-based therapeutics, from their fundamental mechanism of action to the persistent challenges in delivery and manufacturing. While siRNA technology offers a powerful approach to selectively silence disease-causing genes, realizing its full potential requires addressing limitations related to stability, off-target effects, immunogenicity, and tissue penetration. Significant progress has been made in developing sophisticated delivery systems, including lipid nanoparticles and tri-GalNAc conjugates, which enhance targeting and cellular uptake. The clinical translation of siRNA therapeutics, exemplified by approved drugs like patisiran and inclisiran, demonstrates the transformative potential of this modality, particularly for rare genetic disorders and prevalent conditions like hypercholesterolemia. Despite the relatively small number of approved siRNA drugs, ongoing research on optimizing delivery, target selection, and manufacturing processes suggests a promising future for siRNA-based therapies, potentially revolutionizing treatment strategies for various diseases. Clinical successes like patisiran and inclisiran contrast with setbacks like bevasiranib and QPI-1007, emphasizing the need to address inherent limitations. Beyond rare diseases, siRNA attracts interest in ocular, infectious, orphan diseases, and immunotherapy. Advancements in delivery methods, including exploring oral administration and chemical modifications to improve stability and efficacy, are crucial for expanding the clinical utility of siRNA. The ability of siRNA to target specific isoforms, its broad-spectrum antiviral potential, rapid therapeutic response, and the possibility of combinatorial approaches with traditional drugs offer exciting avenues for future development. Continued research and development and lessons learned from successes and failures will be essential to fully realize siRNA’s therapeutic promise.

## Figures and Tables

**Table 1:** Different types of modifications, examples, and impacts on siRNA.

Sugar Modification	Examples	Effects on siRNA
2′-O-Methyl (2′-OMe)	Methyl group at the 2′ position of the ribose sugar. Naturally occurring in some RNAs	Increases stability against nucleases (enzymes that break down nucleic acids), reduces immune stimulation.
2′-Deoxy-2′-Fluoro (2′-F)	Fluorine atom at the 2′ position of the ribose sugar.	Similar to 2′-O-Methyl, enhances stability and can improve target binding and maintain RNA-induced silencing complex (RISC) activity.
2′-O-Methoxyethyl (2′-O-MOE)	2-Methoxyethyl group at the 2′ position of the ribose sugar.	Analog of 2′-OMe with higher RNA binding affinity. Increases nuclease resistance with ΔTm of 0.9–1.7 °C per modification
2′-Arabino-Fluoro (2′-Ara-F)	Fluorine at the 2′ position with a different sugar configuration than 2′F	Alternative fluoro substitution used in siRNA and antisense oligonucleotides
2′-O-Benzyl & 2′-O-CH_2_Py		Compatible with siRNA in vivo. Specific positions enhance activity (e.g., at positions 8 and 15 on the guide strand).
Locked Nucleic Acid (LNA)	Methylene bridge connecting the 2′-O and 4′-C of the ribose, “locking” the sugar in a specific conformation	Bridges the 2′ and 4′ positions of the ribose sugar, dramatically increasing binding affinity to the target mRNA. Can be used sparingly due to potential for increased toxicity.
**Backbone Modifications**		
Phosphorothioate	Replacements of the phosphate backbone	Replaces a non-bridging oxygen in the phosphate backbone with sulfur. Greatly increases nuclease resistance.
Phosphorodithioate (PS2)		Both non-bridging oxygen atoms are replaced by sulfur atoms. Enhances affinity between RISC and siRNA for better activity.
Methylphosphonate (MP) and Methoxypropylphosphonate (MOP)		In MP, one of the non-bridging oxygen atoms is replaced by a methyl group, but in MOP, a methoxy propyl group is replacing an oxygen. Reduce protein binding due to charge-neutral properties. Mitigate hepatotoxicity in ASOs.
Peptide Nucleic Acid (PNA)	Replacement of the ribose phosphate backbone with a peptide backbone	Used for oligonucleotide drug design. Often modifies detection probes for nucleic acid targets.
Phosphonate Analog		Enhance stability and RISC loading. Includes 5′-(E)-vinyl phosphonate (5′-(E)-VP), methylphosphonate, and others. 5′-(E)-VP developed for ss-siRNA by Ionis Pharmaceuticals improves potency and tissue accumulation
**Base Modifications**		
2-thiouridine S_2_U	Can enhance target binding and reduce off-target effects. Enhance the accuracy and efficiency of siRNA translation by stabilizing RNA duplexes and pre-organizing single-stranded RNA for hybridization	
N-Ethylpiperidine triazole	Modify adenosine for reduced immunogenicity. Examples include 7-EAA (forms A-form helix and pairs with uridine).	
6′-Phenylpyrrolocytosine (PhpC)	Cytosine mimic with high fidelity and fluorescence. Useful for monitoring cellular uptake and trafficking.	
2,4-Difluorotolylribonucleoside (rF)	Well tolerated but forms unstable base pairs with adenosine. Reduces off-target activity.	
5-Nitroindole Modification	Reduces passenger strand activity without affecting guide strand effectiveness. Mitigates off-target effects.	
5-Fluoro-2′-Deoxyuridine (FdU)	Triggers DNA-damage pathways and apoptosis. Potential application in siRNA-based cancer therapy	

**Table 2:** GalNac, LNP, and Naked siRNA are different siRNA delivery approaches, each with advantages and characteristics. Here are the key differences between them.

	GalNAc	Lipid-Based	Naked siRNA	References
Tri-GalNAc (N-acetylgalactosamine) Conjugation	Tri-GalNAc is a small sugar molecule that can be conjugated to siRNA molecules. It facilitates the targeting of siRNA, particularly to hepatocytes, which specify a high level of the asialoglycoprotein receptor (ASGPR) on their surface. Tri-GalNAc conjugation enhances siRNA delivery to liver cells, increasing efficacy and reducing off-target effects.	Lipid-based and naked siRNA delivery systems do not rely on tri-GalNAc conjugation for specific targeting of hepatocytes. Instead, they can deliver siRNA to various cell types through different mechanisms.		[81,103,104,105]
Structure	Tri-GalNAc is typically conjugated to the 3′-end of the sense strand siRNA molecule or other delivery vehicles, such as lipid nanoparticles.	Lipid-based siRNA delivery systems contain lipid nanoparticles that encapsulate and protect siRNA molecules. The lipid nanoparticles can be composed of cationic lipids, which help to form stable complexes with siRNA or other lipid components designed to enhance delivery and cellular uptake.	Naked siRNA molecules that are not encapsulated or protected by any delivery system. They are typically administered as free siRNA molecules.	[8,106,107,108,109,110,111,112]
Target cell specificity	Tri-GalNAc conjugation allows for specific targeting of siRNA to hepatocytes in the liver by binding to the ASGPR receptor. This targeting improves siRNA’s cellular uptake and efficacy in liver-related diseases, such as hepatitis and liver cancer.	Lipid-based and naked siRNA delivery systems do not possess inherent target cell specificity, and the cellular uptake and distribution of siRNA rely on factors like nanoparticle properties, formulation, and route of administration. The lipid nanoparticles are often modified by incorporating targeting ligands (e.g., antibodies, aptamers) that specifically bind to receptors on the surface of desired target cells		[106,113,114,115,116,117,118,119,120,121,122]
Stability and cellular uptake efficiency	Chemical modification significantly improves the metabolic stability of siRNA and enhances cellular uptake, specifically in hepatocytes, leading to improved siRNA efficacy in the liver when conjugated to tri-GalNAc for targeted delivery to hepatocytes	Lipid nanoparticles protect siRNA against degradation and facilitate efficient cellular uptake, increasing siRNA stability and higher delivery efficiency.	Naked siRNA is typically less stable and has lower cellular uptake efficiency due to its susceptibility to degradation and limited ability to enter cells.	[123,124,125,126,127]

**Table 3:** Other examples of tri-GalNAc conjugate therapeutic agents in clinical trials [183].

Compounds	NCT Number	Study Title	Study Status	Conditions	Interventions	Sponsor	Phases	Study Type	Completion Date
LY3819469	**NCT05932446**	A Study to Compare Two Formulations of LY3819469 in Healthy Participants	Completed	Healthy	DRUG: LY3819469	Eli Lilly and Company	PHASE1	Interventional	13 November 2023
	**NCT05841277**	A Study of LY3819469 in Participants with Impaired and Normal Renal Function	Completed	Renal Insufficiency Healthy	DRUG: LY3819469	Eli Lilly and Company	PHASE1	Interventional	4 March 2024
	**NCT05936138**	A Study of LY3502970 in Participants with Normal and Impaired Renal Function	Completed	Renal Insufficiency Healthy	DRUG: LY3502970	Eli Lilly and Company	PHASE1	Interventional	21 June 2024
	**NCT04914546**	A Study of LY3819469 in Healthy Participants	Completed	Healthy	DRUG: LY3819469 DRUG: Placebo	Eli Lilly and Company	PHASE1	Interventional	9 November 2022
	**NCT05565742**	A Study of LY3819469 in Participants with Elevated Lipoprotein(a) [Lp(a)]	Completed	Lipoprotein Disorder	DRUG: LY3819469 DRUG: Placebo	Eli Lilly and Company	PHASE2	Interventional	18 October 2024
	NCT06292013	A Study to Investigate the Effect of Lepodisiran on the Reduction in Major Adverse Cardiovascular Events in Adults With Elevated Lipoprotein(a) - ACCLAIM-Lp(a)	Recruiting	Atherosclerotic Cardiovascular Disease (ASCVD) Elevated Lp(a)	Drug: Lepodisiran Sodium Drug: Placebo	Eli Lilly and Company	PHASE 3	Interventional	March 2029
LY3561774	**NCT04644809**	A Study of LY3561774 in Participants with Dyslipidemia	Completed	Dyslipidemias	DRUG: LY3561774 DRUG: Placebo	Eli Lilly and Company	PHASE1	Interventional	17 May 2022
	**NCT05256654**	A Study of LY3561774 in Participants with Mixed Dyslipidemia	Completed	Dyslipidemias Lipid Metabolism Disorders Metabolic Diseases Hyperlipoproteinemia	DRUG: LY3561774 DRUG: Placebo	Eli Lilly and Company	PHASE2	Interventional	3 May 2024
DCR-AUD	**NCT05021640**	Study of DCR-AUD in Healthy Volunteers	Completed	Alcohol Use Disorder (AUD)	DRUG: DCR-AUD DRUG: Placebo for DCR-AUD	Dicerna Pharmaceuticals, Inc., a Novo Nordisk company	PHASE1	Interventional	31 December 2022
	**NCT05845398**	Phase 1b Study of DCR-AUD in Healthy Volunteers	Completed	Alcohol Use Disorder	DRUG: DCR-AUD DRUG: Placebo	Dicerna Pharmaceuticals, Inc., a Novo Nordisk company	PHASE1	Interventional	22 August 2023
ARO-HSD	**NCT04202354**	Study of ARO-HSD in Healthy Volunteers and Patients with Non-Alcoholic Steatohepatitis (NASH) or Suspected NASH	Completed	Non-alcoholic Steatohepatitis	DRUG: ARO-HSD Injection DRUG: sterile normal saline (0.9% NaCl)	Arrowhead Pharmaceuticals	PHASE1	Interventional	23 November 2021
JNJ-75220795	**NCT05039710**	A Study of JNJ-75220795 in Japanese Participants	Terminated	Fatty Liver	DRUG: JNJ-75220795 OTHER: Placebo	Janssen Pharmaceutical K.K.	PHASE1	Interventional	22 February 2023
	**NCT04844450**	A Single and Multiple Ascending Dose Study of Subcutaneously Administered JNJ-75220795	Completed	Fatty Liver Disease	DRUG: JNJ-75220795 DRUG: Placebo	Janssen Research & Development, LLC	PHASE1	Interventional	31 March 2023
ARO-MUC5AC	**NCT05292950**	Study of ARO-MUC5AC in Healthy Subjects and Patients with Muco-Obstructive Lung Disease	Terminated	Asthma Chronic Obstructive Pulmonary Disease	DRUG: ARO-MUC5AC DRUG: Placebo	Arrowhead Pharmaceuticals	PHASE1	Interventional	12 November 2024
ARO-MMP7	**NCT05537025**	Study of ARO-MMP7 Inhalation Solution in Healthy Subjects and Patients with Idiopathic Pulmonary Fibrosis	Recruiting	Idiopathic Pulmonary Fibrosis	DRUG: ARO-MMP7 Inhalation Solution DRUG: Placebo	Arrowhead Pharmaceuticals	PHASE1 PHASE2	Interventional	March 2025
ARO-RAGE	**NCT05533294**	Study of ARO-RAGE in Healthy Subjects	Completed	Asthma	DRUG: ARO-RAGE Injection DRUG: Placebo	Arrowhead Pharmaceuticals	PHASE1	Interventional	8 February 2024
	**NCT05276570**	Study of ARO-RAGE in Healthy Subjects and Patients with Asthma	Active, not recruiting	Inflammatory Lung Disease	DRUG: ARO-RAGE DRUG: Placebo	Arrowhead Pharmaceuticals	PHASE1	Interventional	March 2025
HZN-457	**NCT05565768**	Study to Evaluate HZN-457 in Healthy Volunteers	Completed	Healthy	DRUG: HZN-45 DRUG: Placebo	Horizon Therapeutics Ireland DAC	PHASE1	Interventional	9 August 2023
ARO-C3	**NCT05083364**	Study of ARO-C3 in Adult Healthy Volunteers and Patients with Complement Mediated Renal Disease	Active, not recruiting	C3 Glomerulopathy IgA Nephropathy	DRUG: ARO-C3DRUG: Placebo	Arrowhead Pharmaceuticals	PHASE1	Interventional	June 2025
ALN-APP	**NCT05231785**	A Study to Evaluate the Safety and Tolerability of ALN-APP in Patients With EOAD	Recruiting	Early-Onset Alzheimer Disease	DRUG: ALN-APP DRUG: Placebo	Alnylam Pharmaceuticals	PHASE1	Interventional	July 2025
	**NCT06393712**	A Phase 2 Trial of ALN-APP in Patients with Cerebral Amyloid Angiopathy	Recruiting	Cerebral Amyloid Angiopathy	DRUG: Placebo DRUG: ALN-APP	Alnylam Pharmaceuticals	Phase 2	Interventional	1 November 2029
ALN-XDH	**NCT05256810**	A Study to Evaluate ALN-XDH in Healthy Subjects and Patients with Gout	Terminated	Gout	DRUG: ALN-XDH DRUG: Placebo	Alnylam Pharmaceuticals	PHASE1 PHASE2	Interventional	25 January 2023
ALN-HSD	**NCT05519475**	A Study to Evaluate the Efficacy and Safety of ALN-HSD in Adult Participants with Non-alcoholic Steatohepatitis (NASH) With Fibrosis with Genetic Risk Factors	Recruiting	Nonalcoholic Steatohepatitis	DRUG: ALN-HSD DRUG: Placebo	Regeneron Pharmaceuticals	PHASE2	Interventional	8 September 2027
	**NCT04565717**	A Study of ALN-HSD in Healthy Adult Subjects and Adult Patients with Nonalcoholic Steatohepatitis (NASH)	Terminated	Non-alcoholic Steatohepatitis NASH	DRUG: ALN-HSD DRUG: Placebo	Alnylam Pharmaceuticals	PHASE1	Interventional	21 December 2023
ALN-KHK	**NCT05761301**	A Phase 1/2 Study to Evaluate ALN-KHK in Overweight to Obese Healthy Volunteers and Obese Patients with T2DM	Active, not recruiting	Type 2 Diabetes Mellitus (T2DM)	DRUG: ALN-KHK DRUG: Placebo	Alnylam Pharmaceuticals	PHASE1PHASE2	Interventional	30 April 2025
JNJ-73763989	**NCT05005507**	A Study of JNJ-73763989, Pegylated Interferon Alpha-2a, and Nucleos(t)Ide Analogs in Participants with Chronic Hepatitis B Virus Infection	Terminated	Hepatitis B, Chronic	DRUG: JNJ-73763989 DRUG: PegIFN-alpha-2a DRUG: Tenofovir disoproxil DRUG: TAF DRUG: ETV	Janssen Research & Development, LLC	PHASE2	Interventional	29 December 2021
	**NCT04667104**	A Study of JNJ-73763989, JNJ-56136379, Nucleos(t)Ide Analogs, and Pegylated Interferon Alpha-2a in Virologically Suppressed Participants with Chronic Hepatitis B Virus Infection	Completed	Hepatitis B, Chronic	DRUG: JNJ-73763989 DRUG: Tenofovir disoproxil DRUG: Tenofovir alafenamide (TAF) DRUG: Entecavir (ETV) monohydrate DRUG: PegIFN-alpha2a	Janssen Research & Development, LLC	PHASE2	Interventional	17 April 2023
	**NCT04002752**	A Study of JNJ-73763989 in Healthy Japanese Adult Participants	Completed	Healthy	DRUG: JNJ-73763989 DRUG: Placebo	Janssen Sciences Ireland UC	PHASE1	Interventional	23 August 2019
	**NCT04586439**	A Study of JNJ-73763989 in Healthy Chinese Adult Participants	Completed	Healthy	DRUG: JNJ-73763989	Janssen Research & Development, LLC	PHASE1	Interventional	18 February 2021
	**NCT04439539**	A Study of JNJ-73763989, Pegylated Interferon Alpha-2a, Nucleos(t)Ide Analog (NA) With or Without JNJ-56136379 in Treatment-naive Participants with Hepatitis B e Antigen (HBeAg) Positive Chronic Hepatitis B Virus (HBV) Infection	Completed	Hepatitis B, Chronic	DRUG: JNJ-73763989 DRUG: PegIFN-alpha-2a DRUG: Tenofovir disoproxil DRUG: Tenofovir alafenamide DRUG: JNJ-56136379	Janssen Research & Development, LLC	PHASE2	Interventional	13 February 2024
	**NCT04129554**	A Study of JNJ 73763989 + JNJ 56136379 + Nucleos(t)Ide Analog (NA) Regimen Compared to NA Alone in e Antigen Negative Virologically Suppressed Participants with Chronic Hepatitis B Virus Infection	Completed	Hepatitis B, Chronic	DRUG: JNJ-73763989 DRUG: JNJ-56136379 DRUG: Placebo for JNJ-73763989 DRUG: Placebo for JNJ-56136379 DRUG: Entecavir (ETV) monohydrate DRUG: Tenofovir disoproxil fumarate (TDF) DRUG: Tenofovir alafenamide (TAF)	Janssen Sciences Ireland UC	PHASE2	Interventional	9 June 2022
	**NCT05275023**	An Efficacy and Safety Study of a Combination of JNJ-73763989, Nucleos(t)Ide Analogs (NAs), and a Programmed Cell Death Protein Receptor-1 (PD-1) Inhibitor in Chronic Hepatitis B Participants	Completed	Hepatitis B, Chronic	DRUG: JNJ-73763989 DRUG: PD-1 inhibitor DRUG: Tenofovir Disoproxil DRUG: Tenofovir Alafenamide DRUG: Entecavir	Janssen Research & Development, LLC	PHASE2	Interventional	31 May 2024
	**NCT04535544**	A Study of JNJ-73763989 + Nucleos(t)Ide Analog in Participants Co-Infected With Hepatitis B and Hepatitis D Virus	Active not recruiting	Hepatitis D, Chronic	DRUG: JNJ-73763989 DRUG: Placebo DRUG: Entecavir (ETV) monohydrate DRUG: Tenofovir disoproxil DRUG: Tenofovir alafenamide (TAF)	Janssen Research & Development, LLC	PHASE2	INTERVENTIONAL	6 March 2025
	**NCT04963738**	A Study of JNJ-73763989 in Adult Participants With Renal Impairment	Completed	Renal Impairment	DRUG: JNJ-73763989	Janssen Research & Development, LLC	PHASE1	Interventional	17 October 2022
	**NCT04208386**	A Study to Evaluate the Effect of Hepatic Impairment on JNJ-73763989	Completed	Hepatic Impairment	DRUG: JNJ-73763989	Janssen Sciences Ireland UC	PHASE1	Interventional	20 July 2020
	**NCT03982186**	A Study of Different Combination Regimens Including JNJ-73763989 and/or JNJ-56136379 for the Treatment of Chronic Hepatitis B Virus Infection	Completed	Hepatitis B, Chronic	DRUG: JNJ-73763989 DRUG: Placebo for JNJ-73763989 DRUG: JNJ-56136379 DRUG: Placebo for JNJ-56136379 DRUG: Nucleos(t)ide Analog (NA)	Janssen Sciences Ireland UC	PHASE2	Interventional	26 April 2022
	**NCT04585789**	A Study to Assess Intrahepatic and Peripheral Changes in Immunologic and Virologic Markers in Chronic Hepatitis B Virus Infection	Completed	Hepatitis B	DRUG: JNJ-73763989 DRUG: JNJ-56136379 DRUG: Entecavir (ETV)|DRUG: Tenofovir disoproxil DRUG: Tenofovir alafenamide (TAF) DRUG: PegIFN-alpha-2a (Optional)	Janssen Research & Development, LLC	PHASE2	Interventional	9 January 2024
	**NCT05123599**	A Study of JNJ-73763989, JNJ-64300535, and Nucleos(t)Ide Analogs in Virologically Suppressed, Hepatitis B e Antigen (HBeAg)- Negative Participants with Chronic Hepatitis B Virus Infection	Completed	Hepatitis B, Chronic	DRUG: JNJ-73763989 BIOLOGICAL: JNJ-64300535 DRUG: ETV monohydrate DRUG: Tenofovir disoproxil DRUG: TAF	Janssen Research & Development, LLC	PHASE1	Interventional	26 June 2024
VIR-2218	**NCT04749368**	Study to Investigate the Safety and Efficacy of BRII-835 and BRII-179 Combination Therapy Treating Chronic HBV Infection	Completed	Hepatitis B, Chronic	DRUG: BRII-835 (VIR-2218) BIOLOGICAL: BRII-179 (VBI-2601) with IFN-α BIOLOGICAL: BRII-179 (VBI-2601)	Brii Biosciences Limited	PHASE2	Interventional	24 July 2023
	**NCT05484206**	Effect of Hepatic Impairment on the PK and Safety of VIR-2218 and VIR-3434	Recruiting	Hepatic Impairment|Cirrhosis	DRUG: VIR-2218 DRUG: VIR-3434	Vir Biotechnology, Inc.	PHASE1	Interventional	25 September 2026
	**NCT04412863**	Study of VIR-2218 With or Without Pegylated Interferon Alpha-2a for Treatment of Chronic Hepatitis B Virus Infection	Completed	Chronic Hepatitis B	DRUG: VIR-2218 DRUG: pegylated interferon-alfa 2a	Vir Biotechnology, Inc.	PHASE2	Interventional	25 March 2024
	**NCT04507269**	Study of VIR-2218 in Patients With Chronic Hepatitis B in Mainland China	Completed	Hepatitis B, Chronic	DRUG: VIR-2218 DRUG: Placebo	Brii Biosciences Limited	PHASE2	Interventional	30 September 2021
	**NCT05612581**	A Platform Study to Evaluate Investigational Therapies in Chronic Hepatitis B Infection	Active, not recruiting	Hepatitis B, Chronic	DRUG: VIR-3434 DRUG: VIR-2218 DRUG: TDF DRUG: PEG-IFNα	Vir Biotechnology, Inc.	PHASE1|PHASE2	Interventional	March 2027
	**NCT05970289**	Investigate the Efficacy and Safety of BRII-835 (VIR-2218) and PEG-IFNα Combination Therapy in Chronic HBV Patients	Active, not recruiting	Chronic Hepatitis B Virus Infection	BIOLOGICAL: PEG-IFNα DRUG: BRII-835	Brii Biosciences Limited	PHASE2	Interventional	February 2026
	**NCT03672188**	Study of VIR-2218 in Healthy Subjects and Patients With Chronic Hepatitis B	Completed	Chronic Hepatitis B	DRUG: VIR-2218 DRUG: Placebo	Vir Biotechnology, Inc.	PHASE1|PHASE2	Interventional	3 September 2020
	**NCT04856085**	Study of VIR-2218, VIR-3434, and/or PEG-IFNα in Subjects With Chronic Hepatitis B Virus Infection	Active, not recruiting	Hepatitis B, Chronic	DRUG: VIR-2218 DRUG: VIR-3434 DRUG: PEG-IFNα	Vir Biotechnology, Inc.	PHASE2	Interventional	June 2027
	**NCT05844228**	A Study to Investigate the Effect of Renal Impairment on the PK and Safety of VIR-2218	Recruiting	Renal Impairment	DRUG: VIR-2218	Vir Biotechnology, Inc.	PHASE1	Interventional	21 November 2025
	**NCT05461170**	SOLSTICE: Combination Therapy for the Treatment of Chronic Hepatitis D Infection	Recruiting	Hepatitis D, Chronic	DRUG: VIR-2218 DRUG: VIR-3434 DRUG: NRTI	Vir Biotechnology, Inc.	PHASE2	Interventional	August 2029
	**NCT04891770**	Study to Evaluate the Safety and Efficacy of Selgantolimod (SLGN)-Containing Combination Therapies for the Treatment of Chronic Hepatitis B (CHB)	Completed	Chronic Hepatitis B	DRUG: Tenofovir Alafenamide DRUG: VIR-2218 DRUG: Nivolumab DRUG: Selgantolimod	Gilead Sciences	PHASE2	Interventional	19 July 2024
AB-729	NCT04980482	Open-Label Study of AB-729, Nucleos(t)Ide Analogue and Pegylated Interferon Alfa-2a in Subjects with Chronic Hepatitis B Infection	Active, not recruiting	Chronic Hepatitis b	DRUG: AB-729 DRUG: Peg-IFNα-2a	Arbutus Biopharma Corporation	PHASE 2	Interventional	April 2025
	NCT04820686	A Study Evaluating Treatment Regimens Containing Vebicorvir (ABI-H0731) in Participants with Chronic Hepatitis B Infection	Terminated	Chronic Hepatitis B	DRUG: VBR DRUG: AB-729 DRUG: SOC NrtI	Assembly Biosciences Arbutus Biopharma Corporation	PHASE 2	Interventional	30 March 2023
	NCT04847440	A Study of Safety and Efficacy of ATI-2173 in Combination with Tenofovir Disoproxil Fumarate in Subjects with Chronic Hepatitis B Virus Infection and Subjects with Hepatitis D Virus Coinfection	Terminated	Hepatitis B, Chronic Hepatitis D	DRUG: ATI-2173 DRUG: Viread DRUG: AB-729	Antios Therapeutics, Inc.	PHASE 2	Interventional	1 September 2022
	NCT06277037	Long-Term Follow-up Study for Subjects With CHB Previously Treated With Imdusiran (AB729)	Recruiting	Long Term Follow-up	Other: Non-interventional	Arbutus Biopharma Corporation	Phase 2	Observational	30 October 2029
	NCT06154278	Intrahepatic and Peripheral Responses to Imdusiran (AB-729) in Chronic Hepatitis B	Recruiting	Chronic Hepatitis B	DRUG: Imdusiran (AB-729)	University of Maryland, Baltimore	Phase 2	Interventional	1 July 2027
